# Transcriptome and metabolome reveal redirection of flavonoids in a white testa peanut mutant

**DOI:** 10.1186/s12870-020-02383-7

**Published:** 2020-04-15

**Authors:** Liyun Wan, Yong Lei, Liying Yan, Yue Liu, Manish K. Pandey, Xia Wan, Rajeev K. Varshney, Jiahai Fang, Boshou Liao

**Affiliations:** 1 Key Laboratory of Biology and Genetic Improvement of Oil Crops, Ministry of Agriculture, Oil Crops Research Institute of Chinese Academy of Agricultural Sciences, Wuhan, China; 2 Key Laboratory of Crop Physiology, Ecology and Genetic Breeding, Ministry of Education, Jiangxi Agricultural University, Nanchang, China; 3 Southern Regional Collaborative Innovation Center for Grain and Oil Crops in China, Nanchang, China; 4 College of Agronomy, Jiangxi Agricultural University, Nanchang, China; 5 Center of Excellence in Genomics, International Crops Research Institute for the Semi-Arid Tropics, Hyderabad, India; 6 School of Plant Biology and Institute of Agriculture, The University of Western, Australia, Crawley, WA Australia

**Keywords:** Peanut (*Arachis hypogaea* L.), Seed coat, Hormone, Flavonoid, Transcriptional regulation, Metabolome

## Abstract

**Background:**

Coat color determines both appearance and nutrient quality of peanut. White seed coat in peanut can enhance the processing efficiency and quality of peanut oil. An integrative analysis of transcriptomes, metabolomes and histocytology was performed on *wsc* mutant and its wild type to investigate the regulatory mechanisms underlying color pigmentation.

**Result:**

Metabolomes revealed flavonoids were redirected in *wsc*, while multi-omics analyses of *wsc* mutant seeds and testae uncovered WSC influenced the flavonoids biosynthesis in testa as well as suberin formation, glycolysis, the TCA cycle and amino acid metabolism. The mutation also enhanced plant hormones synthesis and signaling. Further, co-expression analysis showed that FLS genes co-expressed with MBW complex member genes. Combining tissue expression patterns, genetic analyses, and the annotation of common DEGs for these three stages revealed that three testa specific expressed candidate genes, *Araip.M7RY3*, *Aradu.R8PMF* and *Araip.MHR6K* were likely responsible for the white testa phenotype*. WSC* might be regulated expression competition between FLS and DFR by controlling hormone synthesis and signaling as well as the MBW complex.

**Conclusions:**

The results of this study therefore provide both candidate genes and novel approaches that can be applied to improve peanut with desirable seed coat color and flavonoid quality.

## Background

Flavonoids are plant polyphenolic secondary metabolites that share a differentially modified but common three ring chemical structure (C6-C3-C6). Thus, on the basis of R1 and R2 site differentiation substitutions within this C6-C3-C6 ring, flavonoids can be classified into at least ten chemical groups, including flavanones, flavones, isoflavonoids, flavans (flavanols), anthocyanins, and flavonols [1]. Anthocyanins are the most conspicuous class of flavonoids and are responsible for pigmentation in flowers, fruits, seeds, and leaves [2]. Flavonols are the most abundant flavonoids in foods; quercetin, kaempferol, and myricetin are three common isoforms [1]. Flavone is another major class of flavonoids which is commonly conjugated with a sugar group via an O-glycosidic link while some flavones also exist in a C-glycosylated form. In contrast, C-glycosylflavones (also called flavone C-glycosides) comprise an important subgroup of flavonoids that are present in numerous plants [3]; these compounds perform functions in UV protection, caterpillar growth inhibition, pathogen defense, and co-pigments for flower coloration [4, 5].

The flavonoid biosynthetic pathway has been clearly elucidated in model plants; it is well-known that the basic C6-C3-C6 skeleton of these compounds begins with one 4-coumaroyl-CoA and three molecules of malonyl-CoA, catalyzed sequentially by CHS, CHI, F3H and F3’H or F3’5’H to produce dihydroflavonols (dihydroquercetin, dihydromyricetin and dihydrokaempferol). The dihydroflavonols are then converted to anthocyanidins which are colored but unstable pigments by two reactions catalyzed by DFR and LDOX. DFR catalyzes dihydroquercetin, dihydrokaempferol, and dihydromyricetin to leucocyanidin, leucopelargonidin, and leucodelphinidin, respectively, while LDOX converts the oxidation of leucocyanidin, leucopelargonidin, and leucodelphinidin to cyanidin, pelargonidin, and delphinidin, respectively. Finally, the synthesis of colored and stable anthocyanins confers the glycosylation of cyanidin, pelargonidin, and delphinidin via UFGTs. For PAs, a single cyanidin-3-glucoside and delphinidin-3-glucoside are methylated by MTs to produce peonidin-3-glucoside and petunidin- or malvidin-3-glucoside, respectively. LAR and ANR catalyze leucocyanidin and cyanidin to catechin and epicatechin, respectively. Subsequently, POD and PPOD possibly catalyzed the formation of PA polymers via the catechin or epicatechin with leucocyanidin molecules in vacuolar compartments.

Flavonols are another key flavonoid subgroup which plays pivotal roles in testa pigmentation [6]. Flavonol derivatives have been shown to influence anthocyanin-mediated coloration via co-pigmentation effects while FLS catalyzes the conversion of dihydrokaempferol and dihydroquercetin to co-pigment flavonols and thus may influence anthocyanin accumulation levels [2, 7].

A number of flavonoid EBGs including *CHS*, *CHI*, *F3H*, and *FLS* are transcriptionally regulated by the three R2R3-MYB transcription factors MYB11, MYB12, and MYB111 [8, 9]. Known flavonoid LBGs such as DFR, LDOX, ANR, and TT12 are activated by the MBW ternary transcriptional complex [10]. The crucial regulatory roles played by plant hormones in controlling flavonoid metabolic processes have been elucidated in recent years. The plant hormone JA, for example, is known to induce anthocyanin accumulation in a range of species [11–17], while COI1 regulates the expression of transcription factors, including PAP1, PAP2, and GL3 which themselves mediate the ‘late’ anthocyanin biosynthetic genes DFR, LDOX, and UF3GT that modulate JA-induced anthocyanin biosynthesis in *Arabidopsis* [16]. In addition, BR is known to influence JA-induced anthocyanin accumulation by regulating ‘late’ anthocyanin biosynthesis genes; it is thought that this regulation might be mediated by MBW transcriptional complexes [17].

Anthocyanins, proanthocyanidins, and flavonols are all abundant flavonoids found in the seed coats of numerous plant species [18, 19]. These compounds not only influence appearance and nutritional qualities but also perform protective roles in the face of microbial pathogens, insect attacks, and against UV light [19, 20]. Generally, almost all natural flavonoids exist in either O-glycoside or C-glycoside forms in plants. Although, dietary flavonoids O-glycoside attract more attention than C-glycoside, C-glycosylflavonoids in most cases tend to have higher antioxidant and anti-diabetes potential than the corresponding O-glycosylflavonoids. The aim of this study is to present the characterization of a white seed coat peanut mutant. Analyses of testae and the seed metabolomes revealed a reprogramming of flavonoid content in the *wsc* mutant; contents of isoflavones, flavanones, flavones, flavone C-glycosides, and flavonols increased while anthocyanin and PA volumes decreased. The use of transcriptomic profiling revealed down-regulation of a subset of genes involved in flavonoid biosynthesis in developing testae in the *wsc* while key genes involved in flavonol synthesis were enhanced and sucrose and glutamate amino acid metabolism, glycolysis, the TCA cycle, hormone synthesis and signaling were all reprogrammed. The results of this study provide key additional information regarding the mechanisms regulating peanut pigmentation as well as genes that can be utilized for the breeding of white colored testa.

## Results

### Phenotypic variation and flavonoid metabolic features

**Fig. 1:**
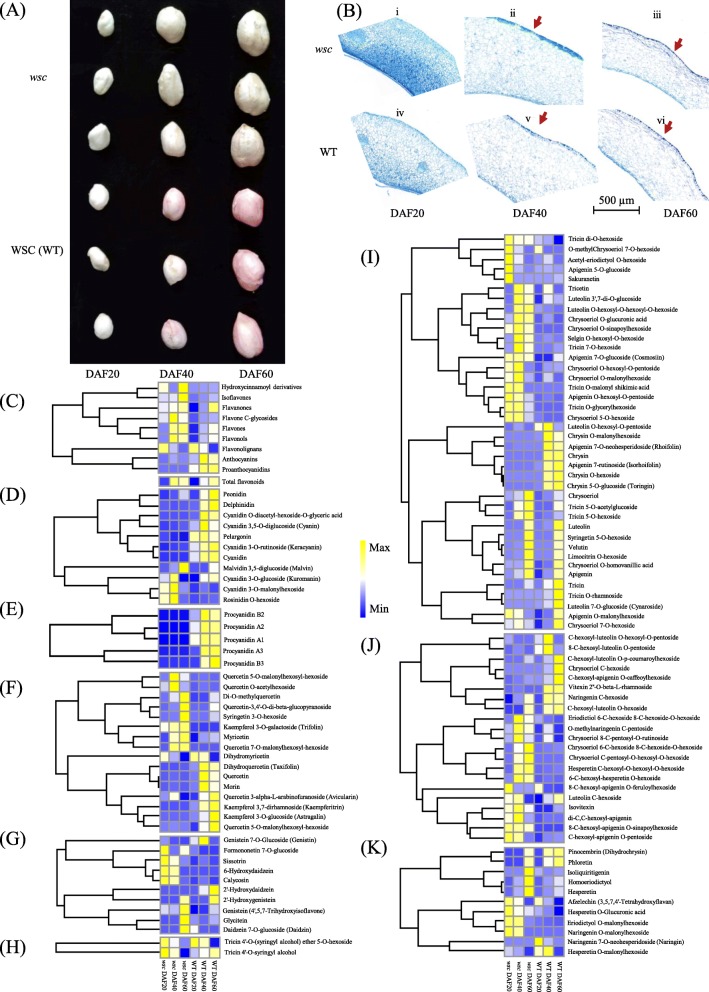
Flavonoids redirected in the testa of *wsc*. **a** Testa color differed between *wsc* and WT during development. **b** Staining of phenolic compounds during seed coat development in *wsc* and WT. **c** Heatmap of total flavonoids and each component contents of total flavonoids between *wsc* and WT. **d** Heatmap of anthocyanins contents between *wsc* and WT. **e** Heatmap of proanthocyanidins contents between *wsc* and WT. **f** Heatmap of flavonols contents between *wsc* and WT. **g** Heatmap of isoflavonols contents between *wsc* and WT. **h** Heatmap of flavonolignans contents between *wsc* and WT. **i** Heatmap of flavones contents between *wsc* and WT. **j** Heatmap of flavone C glycoside contents between *wsc* and WT. **k** Heatmap of flavanone contents between *wsc* and WT. The metabolite contents were scaled using Z-score of peak area (mean value of three biological replications) in the heatmap.

A white seed coat peanut mutant *wsc* was identified from a mutation population derived from an elite pink seed coat variety Zhonghua 16 (cultivated by Oil Crops Reasearch Institute of Chinese Academy of Agricultural Sciences) once treated with ^60^Co. The WT accumulated pink anthocyanins and no obvious color change was observed in *wsc* during development (Fig. 1a). A histochemical analysis was therefore performed to determine the nature of polyphenol compounds in developing testae between *wsc* and WT. TBO staining of developing testae transverse sections revealed the distribution of polymeric phenolic compounds and showed no significant differences between *wsc* and WT in the three seed development stages assessed (Fig. 1b). A series of flavonoid metabolome profiles were therefore conducted between *wsc* and WT. We detected a total of 199 flavonoids in testae samples including 19 anthocyanins, 23 flavanones, 63 flavones, 32 flavone C-glycosides, 38 flavonols, three flavonolignans, one hydroxycinnamoyl derivative, 15 isoflavones, and five proanthocyanidins (Additional file 1). Data revealed that the total flavonoid content in *wsc* was 1.67 times higher than in WT at DAF20, declining to just 1.43 times and 0.97 times that much at DAF40 and DAF60, respectively (Fig. 1c). It is clear that both *wsc* and WT accumulated flavonoids while developing; flavonoid content peaked at DAF40 in *wsc* compared with WT samples at DAF60 (Fig. 1c). Anthocyanins, proanthocyanidins, and flavonols, all the dominate flavonoids, were enhanced throughout development except flavonolignan. The anthocyanins detected in this study included cyanidin, delphinidin, malvidin, pelargonidin, peonidin, rosinidin, and derivatives, data showed that peonidin, delphinidin, cyanidin O-diacetyl-hexoside-O-glyceric acid, cyanidin 3,5-O-diglucoside (cyanin), pelargonin, cyanidin 3-O-rutinoside (keracyanin), and cyanidin levels all increased throughout the development of testae in WT while remained at very low stable levels in *wsc*. Levels of the four glycosylated anthocyanins [i.e., malvidin 3,5-diglucoside (Malvin), cyanidin 3-O-glucoside (Kuromanin), cyanidin 3-O-malonylhexoside, and rosinidin O-hexoside] all increased in *wsc* during the two early developmental stages and then decreased to equal amounts in the WT at DAF60 (Fig. 1d), only malvin keeps the increasing trend in *wsc*. At the same time, procyanidin A1, procyanidin A2, procyanidin A3, procyanidin B1, and procyanidin B2 all occurred at higher levels in WT samples, especially at DAF40 and DAF60, while procyanidin contents in *wsc* remained three orders of magnitude lower (Fig. 1e). The content of flavonols was also redirected over the course of this experiment between *wsc* and WT, and eight of these were up-regulated while nine were down-regulated in *wsc* (Fig. 1f). Indeed, both the majority and total amounts of isoflavones were enhanced in *wsc* (Fig. 1g), while flavonolignan volumes did not change significantly between WT and *wsc* (Fig. 1h). Flavones were also redirected between *wsc* and WT; the total contents of these compounds were surprisingly enhanced in *wsc* (Fig. 1c, i). A total of 13 flavone C glycosides were up-regulated in this analysis while eight were down-regulated in *wsc* although total contents obviously increased overall (Fig. 1c and j). Considerable increases in four flavanones were also seen during the two early developmental stages, three were significantly strengthen at DAF60, while two of the other four differentiated flavanones had declined at DAF60 as well as one at DAF20 and DAF 40, respectively (Fig. 1k).

An untargeted lipid metabolome analysis was also performed as part of this study identifying 6424 differential known metabolites (Additional file 2), KEGG enrichment analysis of differentiated metabolites (Additional file 2) validated the enrichment of flavonols and also showed that isoflavonoid biosynthesis between *wsc* and WT remained quite consistent across the flavonoid metabolome. Differential over-representation was also revealed in several metabolic pathways, including brassinosteroid, cutin, suberin, and wax biosynthesis, as well as arginine, proline, starch, sucrose, and galactose metabolism (Additional file 2).

A seed metabolome analysis was performed to investigate whether, or not, *wsc* influence embryo nutrient quality. A total of 439 metabolics were identified at differential levels within embryos of *wsc* and WT (Additional file 3) with the top five enriched pathways recorded as metabolic pathways, biosynthesis of secondary metabolites, isoflavonoid biosynthesis, flavonoid biosynthesis, and flavone and flavonol biosynthesis (Additional file 4A). Particularly, all differential metabolites from isoflavonoid biosynthesis were up-regulated during this analysis (Additional file 4A). At the same time, five of the six metabolites from flavone and flavonol biosynthesis were enhanced, and the contents of 3,7-Di-O-methylquercetin, quercetin 3-O-[beta-D-xylosyl-(1- > 2)-beta-D-glucoside], and kaempferide in *wsc* rose to more than five times those seen in WT (Additional file 3). All metabolites involved in phenylpropanoid biosynthesis and phenylalanine metabolism declined at different levels (Additional file 3), while both the two involved in cutin, suberin, and wax biosynthesis were down-regulated in *wsc* (Additional file 4A). The results of this analysis are consistent with those from testae flavonoid metabolomes. Testae play crucial role in transporting, as the main energy storage form, fatty acid contents in *wsc* and WT were tested. Results reveal that almost all fatty acid components together with the total fatty acid contents decreased over the course of this study between 5.47–19.41% (Additional file 4B).

### Global transcriptome analysis reveals the involvement of multiple biological processes during testa development

**Fig. 2:**
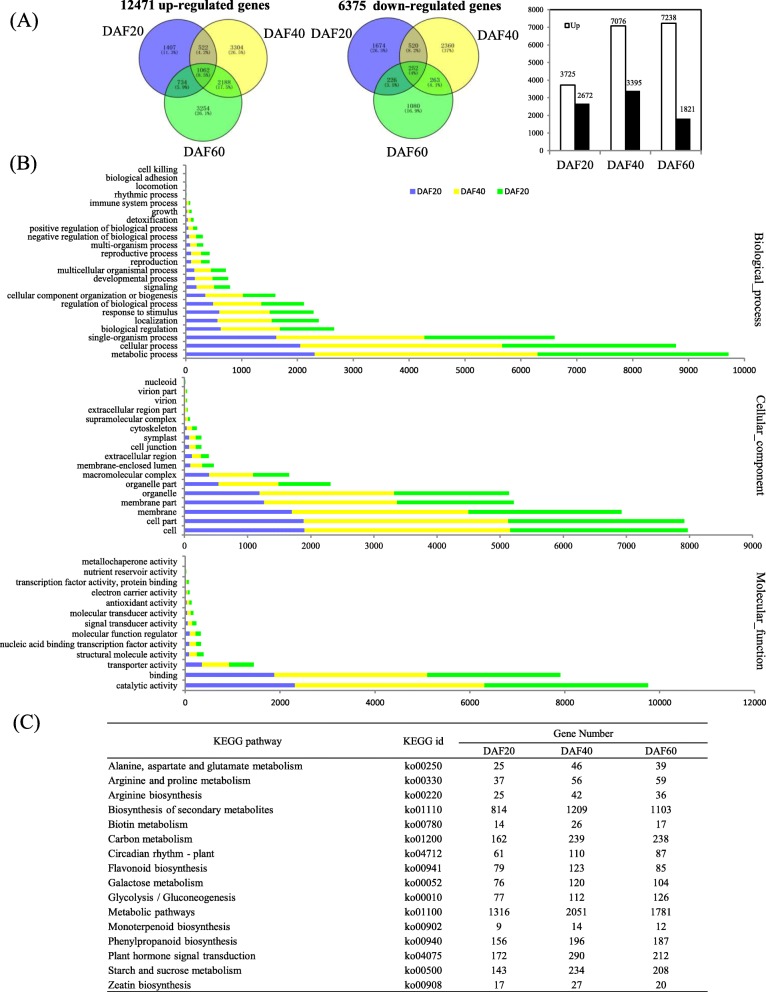
Reprogrammed gene expression in white peanut testae. **a** Venn diagrams displaying overlap between DEGs identified in peanut testae at DAF20, DAF40, and DAF60. The numbers in parentheses showed percentages with respect to the total upregulated and downregulated genes. **b** Selected GO terms enriched among DEGs identified in peanut testae of *wsc* compare with wild types for indicated times. Color panels highlight the three developmental stages assessed in this study. **c** KEGG pathway enrichment analysis among DEGs identified in peanut testae of *wsc* compared with WT for designated DAF period

In order to identify the genes that underlie the formation of white testa phenotype, RNA-seq were conducted on both *wsc* and WT samples collected at DAF20, DAF40, and DAF60. Fold change ≥2 and stringent FDR value ≤0.001 were used as thresholds to identify DEGs. Results revealed a total of 17,428 DEGs between *wsc* and WT (Fig. 2a; Additional file 5). A total of 6397DEGs, 10,471 DEGs, and 9059 DEGs were identified at DAF20, DAF40, and DAF60, respectively (Fig. 2a). Up-regulated genes (3725) presented at DAF20 were significantly more than down-regulated ones (2672), while the number of up-regulated genes (7036) at DAF40 was much more than those down-regulated (3395). 7238 up-regulated genes and 1821 down-regulated ones were uncovered at DAF60 (Fig. 2a).

To analyze the functions of the DEGs between *wsc* and WT, a GO analysis was performed using GOseq method in Blast2GO. Data revealed that metabolic process, cellular process, single-organism process, biological regulation, and localization were all dominant biological process categories (Fig. 2b, Additional file 6). In terms of GO terms relating to cellular component, most DEGs were correlated with five major biological processes, including cell as well as cell part, membrane, membrane part, and organelle (Fig. 2b, Additional file 6). Catalytic activity, binding, transporter activity, structural molecule activity, and nucleic acid binding transcription factor activity were the top categories annotated for the molecular function (Fig. 2b, Additional file 6).

Further, 132, 135, and 134 metabolic pathways of the DEGs associated with the three different developmental stages of the two lines were uncovered by KEGG pathway analysis, respectively. A total list of the metabolic pathways identified in this study is presented in Additional file 7, alongside the top 16 common metabolic and biological pathways identified in the *wsc* compared with WT (Fig. 2c). These results revealed that metabolic pathways were the most enriched, followed by secondary metabolite biosynthesis, plant hormone signal transduction, carbon metabolism, endocytosis, starch and sucrose metabolism, and amino acid biosynthesis (Additional file 7).

### qPCR verification of *wsc* and WT DEGs

To validate the repeatability and reproducibility of gene expression data obtained by RNA-seq, we performed biologically independent qRT-PCR to test the expression level of 17 genes with FPKM values ≥2. The gene-specific primer pairs designed with the Primer Express 3.0 software used for this analysis are listed in Additional file 8. The linear regression analysis revealed a well correlation coefficient (*R*) of 0.84, indicating a strong relationship between the expression data revealed by RNA-Seq and abundances assayed using qRT-PCR (Additional file 9).

### WSC mutation influences flavonoid metabolic pathways

**Fig. 3:**
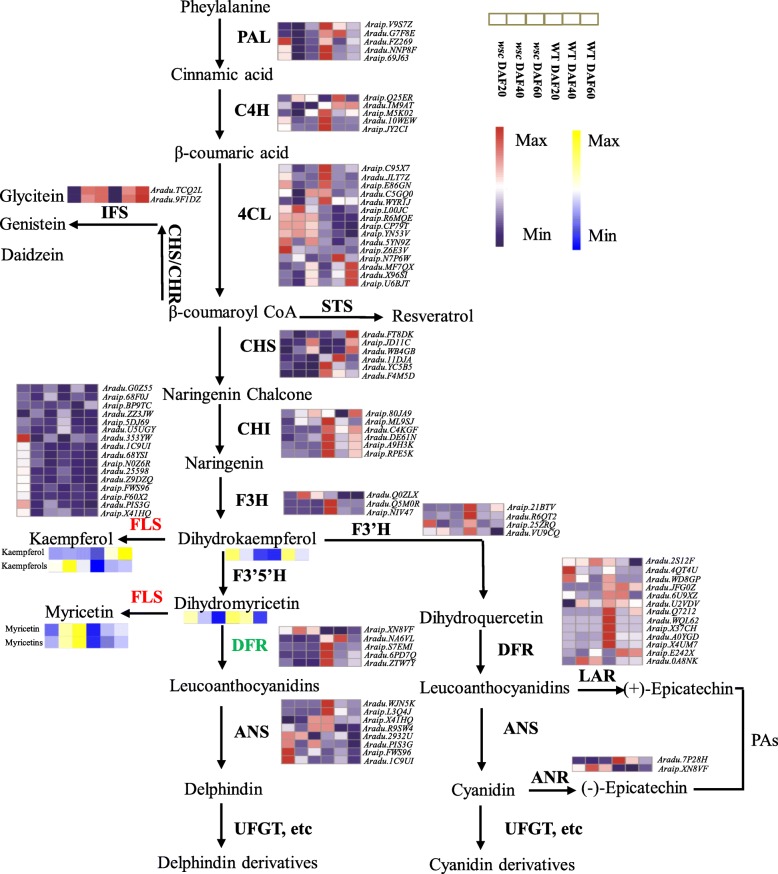
Flavonoid/anthocyanin pathways involved in peanut seed coat development. PAL, phenylalanine ammonia lyase; C4H, cinnamate 4-hydroxylase; 4CL, 4-coumarate:CoA ligase; CHS, chalcone synthase; CHI, chalcone isomerase; CHR, chalcone reductase; F3H, flavanone 3-hydroxylase; IFS, 2-hydroxyisoflavanone synthase; F3’H, flavonoid 3′-hydroxylase:flavonoid 3′5′-hydroxylase; FLS, flavonol synthase; DFR, dihydroflavonol 4-reductase; LAR, leucoanthocyanidin reductase; ANS, anthocyanidin synthase; ANR, anthocyanidin reductase; UFGT, anthocyanidin 3-*O*-glucosyltransferase. Gene expression was scaled using Z-scores of FPKM for mean valued of three biological replicates in heatmaps

Flavonoid components determine the color of the peanut testa [21–23]. Flavonoid biosynthesis pathway genes were searched based on their KO identifications within the KEGG database as well as synonyms identified in combined with functional annotations, this enabled the identification of 433 DEGs in flavonoid metabolic pathways (Table 1). The genes involved in the three secondary metabolic pathways (i.e., flavonoid, anthocyanin, and flavones/flavonol biosynthesis) related to pigmentation were analyzed in this study using testa transcripts, the core genes in these pathways were studied in detail, and results demonstrated that most exhibited significant changes in expression levels over the course of this analysis. Indeed, regardless of whether these were EBGs (e.g., *CHI*) or LBGs (e.g., *ANS*, *UFGT*), all exhibited higher transcript abundances within WT compared to *wsc* with the exception of 4-coumarate: CoA ligase genes and their flavonol synthase counterparts (Fig. 3). As a branch point in flavonoid biosynthesis, the dihydroflavonols are the intermediates in the production of both the colourless flavonols and the colored anthocyanins through FLS and DFR, respectively. Anthocyanins contents were decreased in *wsc*, which corresponds to lower level expression of *DFR* genes. While, myricetins and kaempferols contents were increased in *wsc*, this consistent with *FLS* expression (Fig. 3). Combining the transcriptome and metabolome information, it could be extrapolated that *DFR* might be the target gene for the loss of pink color in *wsc*. The competition between FLS and DFR for common dihydroflavonols substrates might obviously block anthocyanins synthesis and leading to the enhanced production of flavonols such as myricetin and kaempferol, which shift the flavonol: anthocyanin ratio in *wsc*.

**Table 1:** Flavonoid pathway genes related to testa pigmentation of peanut

Function	Gene	Enzyme	KO id (EC.no)	No.All	DEGs in DAF20	DEGs in DAF40	DEGs in DAF60
	*PAL*	Phenylalanine ammonia-lyase	K10775	14	2	5	0
	*C4H*	Trans-cinnamate 4-monooxygenase	K00487	6	5	2	3
	*4CL*	4-coumarate--CoA ligase	K01904	45	8	15	6
Anthocyanin biosynthesis	*CHS(STS)*	Chalcone synthase(Stilbene synthase)	K00660	76	11	43	18
	*CHI*	Chalcone isomerase	K01859	21	5	5	3
	*F3H*	Flavanone 3-hydroxylase	K00475	9	2	5	4
	*F3’H*	Flavanone 3′-hydroxylase	K05280	11	0	1	0
	*DFR*	Dihydroflavonol 4-reductase	K13082	17	5	6	5
	*ANS*	Anthocyanidin synthesis	K05277	20	6	4	9
	*UFGT*	Anthocyanidin 3-O-glucosyltransgersae	K12930	5	0	0	0
Anthocyanin modification	*UGT75C1*	Anthocyanidin 5-O-glucosyltransgersae	K13692	52	12	7	3
	*GT1*	Anthocyanidin 5,3-O-glucosyltransgersae	K13263	6	0	3	0
	*FLS*	Flavonal synthase	K05278	70	14	18	14
	*FOMT*	Flavonol 3-O-methyltransferase	K05279	39	3	3	5
	*UF3GT*	flavonol 3-O-glucosyltransferase	K12930	5	0	0	0
Flavanone biosynthesis	*ANR*	anthocyanidin reductase	K08695	3	2	2	2
	*LAR*	leucoanthocyanidin reductase	K13081	34	10	9	9

### Suberin in *wsc* testa epidermis

**Fig. 4:**
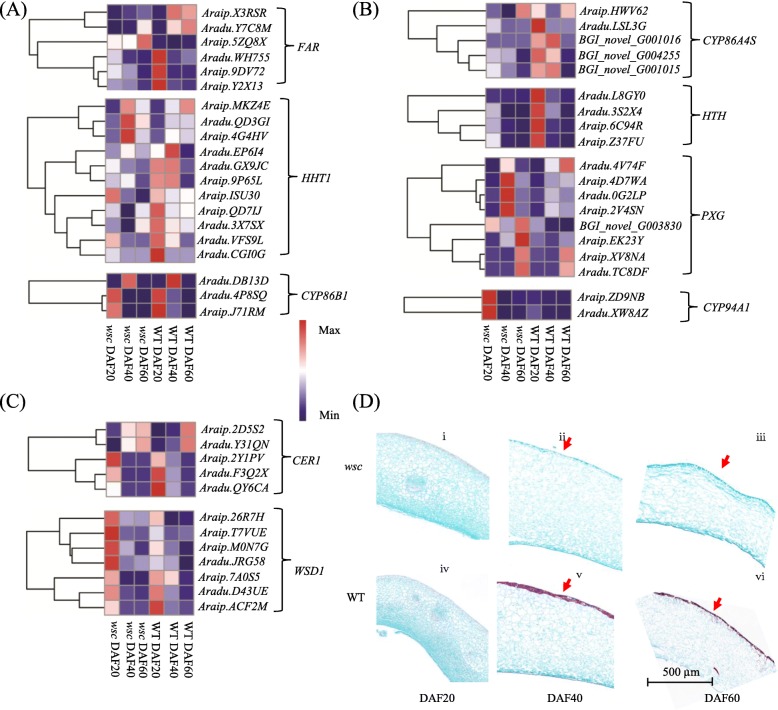
Changes in wax, cutin, and suberin synthesis during testae development in *wsc* and WT. **a** Expression of suberin synthesis pathway genes. **b** Expression of cutin synthesis pathway genes. **c** Expression of glutamate amino acid synthesis pathway genes. **d** Changes of suberin compounds during seed coat development in *wsc* and WT. FAR, alcohol forming fatty acyl-CoA reductase; ASFT/HHT, aliphatic suberin feruloyl transferase; CYP86B1, fatty acyl hydroxylase (cytochrome P450 monooxygenase); CER1, ECERIFERUM1; WSD1, wax synthase/acyl-CoA:diacylglycerol acyltransferase; CYP86A4S, cytochrome P450s; HTH, HOTHEAD (glucose-methanol-choline <GMC> oxidoreductase); PXG, peroxygenase; CYP94A1, cytochrome P450 CYP94A1. Gene expression was scaled using Z-scores of FPKM for mean valued of three biological replicates in heatmaps

Suberin and its associated waxes play important roles in plant development [24]. As flavonoids in the testa of *wsc* changed greatly over the course of this analysis, KEGG pathway involved in cutin, suberin, and wax biosynthesis were further analyzed in this study. Data indicated that most cutin and suberin synthesis pathway genes showed significantly higher levels of gene transcripts in *wsc* (Fig. 4a and b) while the genes involved in wax synthesis exhibited no obvious regularity (Fig. 4c). Five of the six *FAR* gens involved in the synthesis of aromatic suberin monomers for the aliphatic domain [25] were significantly depressed at either DAF20 or DAF60, eight of 11 *HHT1* DEGs were up-regulated, and all *CYP86B1* genes exhibited a higher level of expression in *wsc* (Fig. 4a). Four out of five *CYP86A4S* genes were classified as DEGs while all four *HTH* genes exhibited enhanced expression levels compare with WTs (Fig. 4a). Suberin staining revealed the presence of almost no red staining in all three developmental stages of *wsc* compared with the deeper red staining in WT during development (Fig. 4d).

### Primary metabolic differences

Transcriptome analysis revealed that the glycolysis and gluconeogenesis pathway was differentially regulated between the *wsc* and WT. The expression of coding genes for the 13 enzymes catalyze the 14-step reactions in glycolysis was also enhanced to varying degrees (Additional files 10, 11). All nine DEGs in hexokinase which function as one of the three key enzymes (i.e., hexokinase, phosphofructokinase, and pyruvate kinase) within the glycolysis pathway markedly increased in expression, especially in the two later stages (Additional files 10A, 11A), while the six DEGs in phosphofructokinase exhibited higher expression levels in *wsc* via different patterns (Additional files 10A, 11B). Two of the 12 pyruvate kinase genes were down-regulated in the *wsc*, especially during the early stage at DAF20, including the highest expressed gene, *Araip.VA90H*. The remaining ten pyruvate kinase genes also exhibited enhanced expression in the mutant (Additional files 10A, 11C).

The products of the glycolysis pathway were then catalyzed by the pyruvate dehydrogenase complex and entered into the TCA cycle. As data revealed that 19 DEGs, 26 DEGs, and 37 DEGs were enriched in this cycle at DAF20, DAF40, and DAF60, respectively, the ten enzyme encoding genes that control this process were analyzed in detail and six were found to be differentially expressed in the *wsc*. Four pyruvate dehydrogenase genes were up-regulated in all three developmental stages in the *wsc*, while the other three were only enhanced at DAF40 (Additional files 10B, 11D). At the same time, two citrate synthase genes increased and one deceased in the *wsc* (Additional files 10B, 11E), while all aconitase genes were characterized by activated expression in this mutant, especially the three high expressed genes (*Aradu.S0KU9, Aradu.83N8C,* and *Araip.SB6JF*) (Additional files 10B, 11F). Isocitrate dehydrogenase expression was also universally higher in the *wsc*, reaching its highest level at DAF40 (Additional files 10B, 11G), while both DEGs in succinate dehydrogenase differed at DAF40 with one activated and the other one depressed (Additional files 10B, 11H). Five of the six malate dehydrogenase genes were up-regulated at either DAF40 or DAF 60 (or both), while *Araip.M2TZZ* was down-regulated in all the three *wsc* developmental stages (Additional files 10B, 11I).

**Fig. 5:**
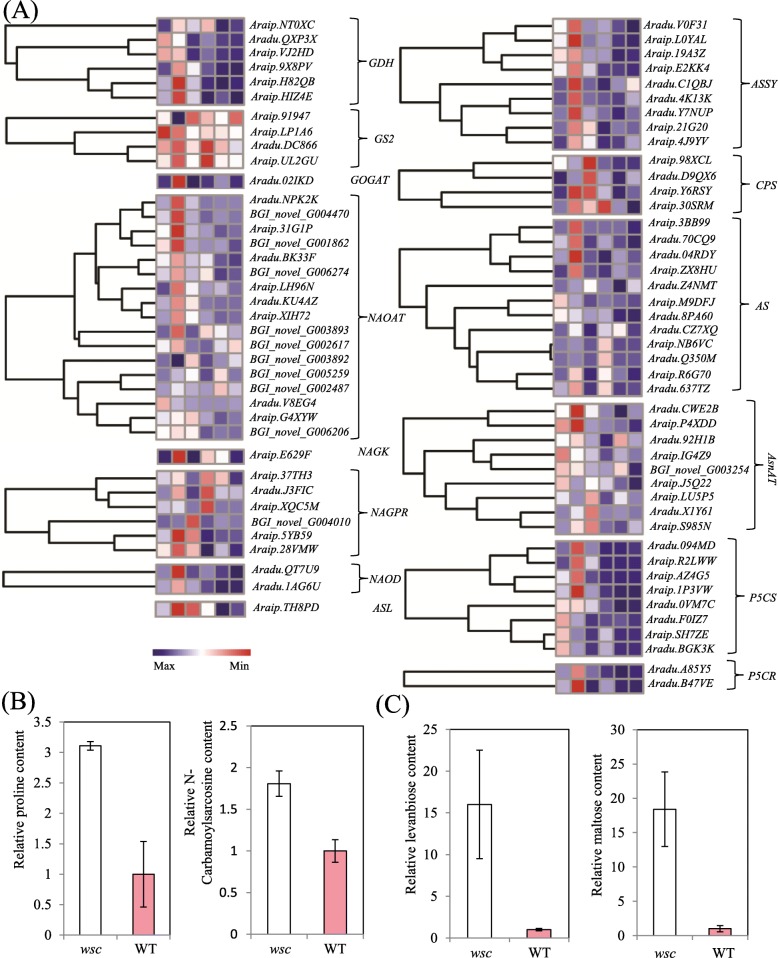
Expression of glutamate amino acids synthesis pathway genes and altered amino acid/sugar contents in *wsc* and WT. **a** Expression of glutamate amino acids synthesis pathway genes. **b** Proline and N-carbamoylsarcosine content in *wsc* and WT. **c** Levanbiose and maltose content in *wsc* and WT. GDH, glutamate dehydrogenase; GS2, glutamine synthetase; 2, GOGAT, glutamate synthase; NAOAT, N-acetylornithine aminotransferase; NAGK, N-acetylglutamate kinase; NAGPR, N-acetylglutamatyl-5-P reductase; NAOD, N-acetylornithine deacetylas; ASL, argininosuccinate lyase; ASSY, argininosuccinate synthase; CPS, carbamoyl phosphate synthetase; AS, asparagine synthase; AsnAT, aspartate aminotransferase; P5CS, delta-1-pyrroline-5-carboxylate synthetase; P5CR, pyrroline-5-carboxylate reductase. Gene expression was scaled using Z-scores of FPKM for mean valued of three biological replicates in heatmaps

DEG investigations within amino acid metabolism pathways revealed that glutamate amino acid synthesis occurred at significantly higher expression levels within *wsc* (Fig. 5a). Five of *P5CS (*one of the two key enzyme coding gene in proline synthesis*)* genes were all characterized by stronger levels of expression at all three developmental stages while the other three had higher expression levels in the *wsc* at the two early stages. Two *P5CR (*another key enzyme coding gene in proline synthesis*)* gene were detected in differential expression; *Aradu.A85Y5,* was characterized by a significantly higher expression level at all three stages while its counterpart, *Aradu.B47VE*, was only activated at DAF20 and DAF40 (Fig. 5a)*.* Metabolome analysis of testae revealed that the proline content in *wsc* was three times that of WT while the N-Carbamoylsarcosine content reached 1.8 times that level when compared with WT (Fig. 5b).

Sugars supply plants with energy and functioned in flavonoids accumulation [26, 27]. Three gene families encoding sucrose transporters have been implicated in plant sugar accumulation, including TSTs [28], SUTs [29–31], and clade III of SWEET [32–34]. We compared the expression patterns of these gene families between *wsc* and WT (Additional file 12) and found seven SUTs to occur at significantly higher levels within *wsc* than WT even though expression patterns were different; *Aradu.M7BX0*, *Aradu.QA3D9*, and *Aradu.SQ8X7* were all owned highest levels at the early developmental stage and adjusted to a moderate level; in contrast, *Araip.53MMF* exhibited a higher expression level within *wsc* at DAF60 while the other three SUTs were characterized by consistently higher expression levels in mutants at the all three stages (Additional file 12). Nine of the 13 DEGs in SWEETs were enhanced and four decreased in expression (Additional file 12). In the case of the monosaccharide transporter, expression of the two STPs in *wsc* was dramatically higher than in WT (Additional file 12). The metabolome profile data elucidated that *wsc* testae included more than ten-fold increases in maltose and levanbiose contents compare with WT (Additional file 12C).

### The vital roles of phytohormones and TFs in the white testa phenotype of *wsc*

Hormone and transcription factors have both been reported to perform functions in flavonoid accumulation [15, 35]. Genes involved in hormone synthesis and signal transductions were compared in this study. Results revealed the presence of five DEGs involved in the BR synthesis pathway, of which three *BR6OX1* were up-regulated either at DAF20 or at DAF40 and DAF60; in contrast, the two *BAS1* genes which reduce the level of active BRs were significantly down-regulated in *wsc* at DAF60 (Additional file 13A). Analysis of the testae metabolome uncovered 16 metabolites within the BR biosynthesis pathway, and all of them were up-regulated (Additional file 2). At the same time, 16 DEGs involved the GA synthesis pathway including *KAO* (3), *GA2ox* (3), *GA3ox* (1), and *GA20ox* (9) were identified in this analysis; the majority of these genes were up-regulated in *wsc* with the exception of *Aradu.BUI3V, Araip.KVM2C,* and *Aradu.U5UGY* that all exhibit opposite expression patterns (Additional file 13B). Further, testae metabolome analysis also supported the enhancement of GA synthesis by identifying three pathway products that increased in content within *wsc* (Additional file 2). While DEGs involved in JA biosynthesis included two *PLA1* (i.e., *BGI_novel_G004010* and *Aradu.Y096Z*), one *AOC* (*Aradu.H2RVW*), four *OPR* (i.e., *Aradu.FV6YV, Araip.F1ZZD, Aradu.0C0YF,* and *Araip.22RGE*), and three *MFP* (i.e., *Araip.UPC07, Araip.M6X0I,* and *Araip.60KBF*) were all enhanced in *wsc* (Additional file 13C). These expression and metabolome data taken together therefore supported the enhancement of BR, GA, and JA synthesis pathways.

A series of DEGs involved in seven hormone signal transduction pathways were also identified in this study, including nine in ABA, 12 in AUX, five in BR, ten in CTK, seven in GA, ten in JA, and four in SA (Additional file 14). Seven ABA signaling DEGs were enhanced, including SRK2 gene Aradu.SKS95 that exhibited the highest FPKM value (Additional file 14A). The vast majority of AUX signaling pathway genes increased in expression in *wsc*; only two deviated from this pattern, *Araip.TU273* and *Araip.60Q0E*, both of which exhibited obvious decrease in mutants at DAF20 (Additional file 14B)*.* Further, four out of five DEGs involved in BR signaling were highlighted in *wsc* (Additional file 14C); and nine of the ten DEGs involved in JA signal pathway were up-regulated and all of them were MYC2 factors (Additional file 14D). Expression patterns and FPKM values of DEGs in hormone signal transduction pathways provided collective evidence of ABA, AUX, JA, and BR signaling enhancements (Additional file 14).

Flavonoid production is transcriptionally regulated by MYB factors and the MBW complex. A total of 24 R2R3-MYB (nine up and 15 down), 26 bHLH (17 up and nine down), and 21 WD40 (19 up and two down) genes were identified in this analysis (Additional file 15) including homologs (*Aradu.CA8XJ* and *Araip.MHR6K*) of *AtTT8* which enables strong, cell-specific accumulation of flavonoids in *Arabidopsis thaliana* [36] and homologs of *AtMYB5* (i.e., *Aradu.JK51Z* and *Aradu.WZF00*) control outer seed coat differentiation alongside *TTG1* and *TT2* [37].

### White testa phenotype candidate genes revealed by multi-omics analysis

In order to identify candidate genes controlling the white testa mutant phenotype in peanut, we analyzed DEGs in common between WT and *wsc* at the three different developmental stages. This analysis resulted in the identification of 1646 unigenes (Fig. 2 and Additional file 16). Observations across whole growth stages and tissues revealed no obvious differences between *wsc* and WT with the exception of the seed phenotype (Additional file 17), therefore *WSC* was identified as a seed-specific gene. Previously published data [38] was interrogated to identify seed specific expressed common DEGs between *wsc* and WT which resulted in 86 candidates genes for the testa phenotype (Additional file 18). The *wsc* was identified in the M3 generation of the mutant population while the segregation ratio of the mutant line between WT and *wsc* was nearly 3:1 (156:49). Combining seed specific expressed common DEGs with the results of the genetic analysis revealed the putative candidate genes *Araip.M7RY3* (*CSN1)*, *Aradu.R8PMF (MYB)* and *Araip.MHR6K (bHLH)* (Additional file 18). The FPKM value of the *Araip.M7RY3* gene in the WT seed coat decreased from 2.15 (DAF20) to 1.15 (DAF40) before falling further to 0.19, while the value for this gene in *wsc* increased from 7.55 (DAF20) to 12.21 (DAF40) and then rose further to 12.84 (DAF60). It is annotated that Araip.M7RY3 is homolog of a COP9 signalosome complex subunit 1(CSN1) encoding gene. Previous studies have revealed that several subunits of the COP9 signalosome complex are involved in regulating flavonoids and that phenylalanine metabolism further regulates proanthocyanidin biosynthesis [39–41]. Aradu.R8PMF and Araip.MHR6K are components of MBW complex which are widely reported function in flavonoids metabolism (Li, 2014)*.* To investigate the differences of the sequences of the three candidate genes between the WT and the mutant plants, we checked SNPs called from the transcriptome data and verified the SNPs by the direct sequencing of the PCR products. As a result, we discovered one SNP in the DNA binding domain of bHLH-MYC_N (pfam14215) of *Araip.MHR6K* (Araip.B06_2335496^[A/G]^) leadting to a 50^T^ to 50^I^ amino acid change which was confirmed by sequencing (Additional file 19). The information that these genes cause a white seed color phenotype require further confirmation in future functional genomics studies.

## Discussion

Appropriate pigmentation determines the appearance and nutrient quality of peanuts. Complex transcriptome, metabolome regulation, and cytological changes therefore suggested that secondary metabolism pathways like flavonoid biosynthesis as well as primary metabolism pathways like glycolysis and gluconeogenesis represented clear differences between *wsc* and WT. Flavonoid synthesis-related genes as well as their counterparts involved in key enzyme coding genes in glycolysis and the TCA cycle, glutamate amino acid synthesis pathway genes, and sugar synthetase and transporter genes differentially expressed between *wsc* and WT. These pathways might well be regulated by upstream phytohormone signals (AUX, BR, and JA) and transcription factors (MYB, bHLH, and WD40). The results provided informative clues that augment understanding of the regulatory network that underlies testa coloration and will therefore contribute to the genetic breeding of ideal quality peanuts.

### Multilevel regulation of the WSC gene

The gene enrichment analysis reported here revealed that 23, 17, and 13 biological processes, cellular component and molecular function mainly associated with metabolism and environmental information processing differentially altered (Fig. 2b and Additional file 6). Genes associated with metabolic process comprised 36.08, 38.09, and 37.71% (i.e., 2308/6397, 3988/10471, and 3416/9059) of total DEG numbers (Additional files 5 and 6), while metabolic pathways, the biosynthesis of secondary metabolites, plant hormone signal transduction, and carbon metabolism were the major biological processes influenced by *wsc* (Fig. 2b). Genes associated with metabolism exhibited a mixed trend, while hormone responses and biosynthesis processes were effectively up-regulated in *wsc*.

**Fig. 6:**
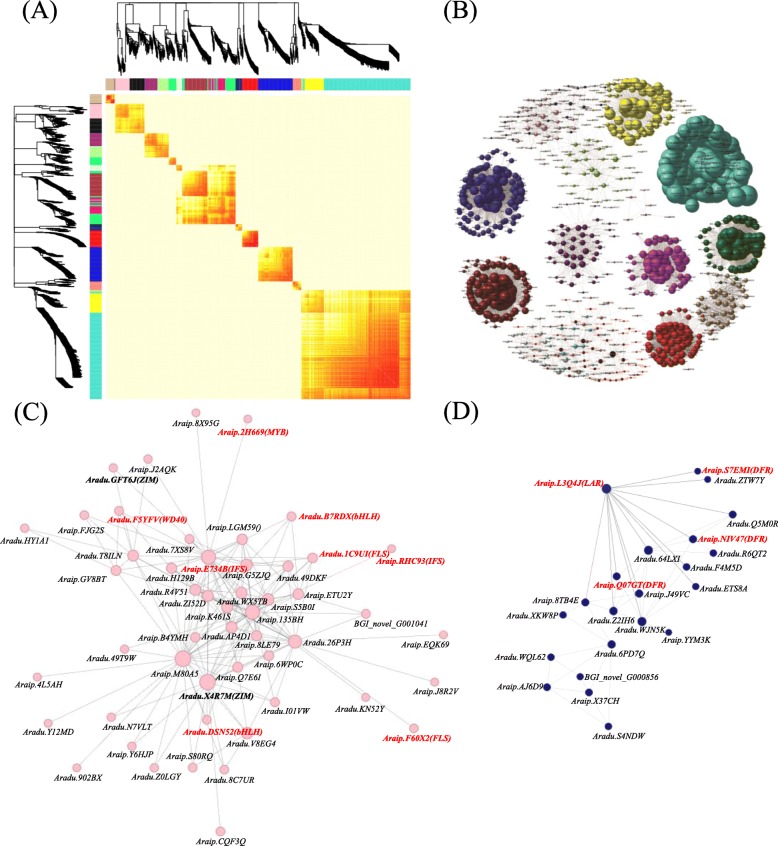
Co-expression of DEGs between *wsc* and WT. **a** Heatmap of co-expressed genes. **b** Network of co-expressed modules. **c** Co-expression module including *FLS* genes. **d** Co-expression module including *DFR* genes

The DEGs detected in this study were classified into 16 co-expression modules, each of which contains representatives that harbor similar expression patterns and might therefore have parallel functions (Fig. 6a, b). KEGG enrichment revealed that 14 co-expressed DEG modules were all enriched in flavonoid biosynthesis, plant hormone synthesis, plant signal transduction, and carbon metabolism. Multi-omics analysis implying the competitive expression of *FLS* and *DFR* genes resulted in the redirect of flavonols and anthocyanin accumulations. Additional emphasis was then placed on modules containing both FLS and DFR genes. Surprisingly, we found FLS genes were co-expressed with MYB genes (*Araip.2H669*), WD40 (*Aradu.F5YFV*), AOC (*Araip.Q7E6I*, *Aradu.T8ILN,* and key enzymes coding genes in the JA synthesis pathway), JAZ (*Aradu.X4R7M* and *Aradu.GFT6J*), MYC2 (*Aradu.DSN52*, *Aradu.B7RDX*, and *Araip.LGM59*) and IFS (*Araip.RHC93* and *Araip.E734B*), indicating the regulation of the FLS genes by MBW complex and the JA signal pathway (Fig. 6c). At the same time, DFR genes were co-expressed with CHS (*Aradu.F4M5D*), F3H (*Araip.NIV47* and *Aradu.Q5M0R*), ANR (*Araip.8TB4E*) and LAR (*Araip.X37CH* and *Aradu.WQL62*) genes (Fig. 6d). It has been reported that both anthocyanin and procyanidine synthesis is regulated by the MBW complex in *Zea mays*, *Antirrhinum majus*, *Petunia hybrid*, and *A. thaliana* [37, 42–46]; although the results of this analysis are inconsistent with above studies, they are nevertheless consistent with the results of peanut pigment regulation research which has shown that anthocyanin biosynthesis is mainly regulated by AtMYB111 homologs (i.e., *c35101_g4* and *c37398_g2*) through EBGs instead of through the MBW complex via LBGs. The results of this study therefore highlight the distinct regulation patterns in peanut testa pigments, possibly from the trait of flower aerially and producing pods underground.

### WSC redirects the phenylpropanoid metabolic flux in peanut testa

Flavonoids are secondary metabolites that accumulate in plants and promote seed and pollen dispersal by contributing to the formation of color in fruits and flowers [47]. Indeed, when the *wsc* was first observed, it was thought that flavonoid content must be markedly decreased compared with WT for the white color, however, that TBO staining of transverse developing testae sections present no huge differences in *wsc* seeds stained colors while metabolome analysis revealed that isoflavones, flavanones,flavones, flavone C-glycosides, flavonols were all significantly increased while anthocyanins and proanthocyanidins contents markedly decreased. Flavonoid C-glycosides have recently been shown to exhibit significant antioxidant activity as well as anticancer and antitumor potential, hepatoprotective, anti-inflammatory, anti-diabetes, antiviral, antibacterial, and antifungal activities as well as other biological effects [48]. It appears that C-glycosylflavonoids in most cases exhibit higher antioxidant and anti-diabetes potential than their corresponding O-glycosyl flavonoids and aglycones [48]. Redirected in flavonoid components, especially the significantly higher flavone C-glycosides and flavonols contents, combined with a very high yield potential imply broad and extensive prospective applications for *wsc*.

### The crucial roles of phytohormones in the formation of a white testa phenotype

Previous studies have elucidated the role of phytohormones in the regulation of flavonoid pathways. MeJA is a phytohormone which plays a key role in plant growth as well as in many physiological and biochemical processes [49]. This compound has been used to stimulate secondary metabolite production in numerous plant species. MeJA enhances antioxidant activity and flavonoid content in blackberries as well as strawberries and olive fruits [50–52]. JA synthesis in the α-linolenic acid pathway was significantly up-regulated in both transcriptomes (i.e, all ten DEGs in the JA synthesis pathway) and metabolomes (i.e., all 16 differential metabolites were up-regulated) data sets in *wsc* (Additional file 13C). JAZs interacted with COI1 and then were degraded through the 26S proteasome to release the downstream transcription factors such as MYC2 to activate the JA responses [53]. In this study, nine of 10 DEGs (MYC2 genes) were up-regulated in *wsc*. Two MYC2 genes correlated with FLS, IFS, and MSW complex were identified by the gene co-expression analysis. MYC2 modulates biosynthesis of indole glucosinolates and tryptophan, resistance to necrotrophic pathogens (e.g., *B. cinerea*), and the expression of *ERF1*, *ORA59*, and *PDF1.2* by regulating JA-dependent responses [15, 53–59]. The multiple functions of MYC2 might be one reason for flavonoid reprogramming in *wsc*.

The biosynthesis of brassinolide from campesterol occurs via two alternative routes in *Arabidopsis*, the first through (6α)-hydroxycampestanol and the second via 6-deoxycathasterone. The enzymes identified in *Arabidopsis* include DWF4, DET2, CPD, BR6OX, and BAS1 [60]. The members of one key gene family, *BR6OX1*, were up-regulated more than two-fold, while the negative regulators of BR biosynthesis, two *BAS1* genes, were depressed in mutants. The expression levels of several key BR signaling and response genes, including *BRI1* (*Araip.0ZF73*, *BGI_novel_G003679*), *BSK* (*Aradu.NG7HH*), and *BAK1 (BGI_novel_G006285)* were up-regulated by more than two-fold. It is well known that BR signaling inhibits BR biosynthesis through BES1 and BZR1 inhibition of the expression of *DWF4*, *CPD* and other biosynthesis genes [61–64]. This might be one reason why obvious BR synthesis enhancements cannot be determined.

Recent studies have also shown that IAA directly interacts with the F-box protein TIR1 and promotes the degradation of Aux/IAA transcriptional repressors to activate diverse auxin responsive genes. The results of this analysis revealed that AUX synthesis was not influenced in the *wsc* while signal transduction was obviously enhanced. The highest expressed *AUX1* gene, *Araip.TU273,* was up-regulated in DAF20 while almost all AUX response genes were induced in the *wsc*. It has been suggested that AUX production in the endosperm drives seed coat development while fertilization of the central cell results in the production of this compound and most likely its export to maternal tissues; this process drives seed coat development by removing PcG function [65]. Activated AUX signaling might be due to strengthened AUX synthesis in the endosperm or transport from endosperm to the testa.

**Fig. 7:**
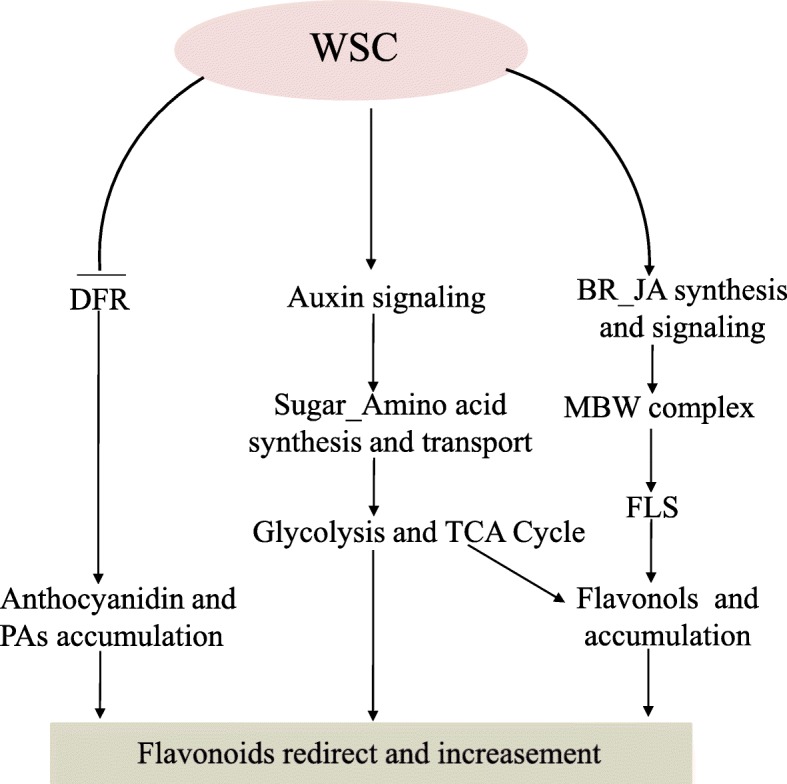
Model to show the mechanism by which WSC regulates the reprogramming of flavonoids in peanut. This model involves WSC as a key factor that positively regulates flavonol and JA biosynthesis and negatively regulates anthocyanidin and PA accumulation. In addition to flavonoids biosynthesis genes, WSC also regulates the expression of several genes associated with auxin signaling, sugar, and amino acid metabolism. This model shows that WSC is an integrator of primary and secondary metabolism

This study describes a peanut mutant with a white testa that contains higher levels of flavonols and flavone C-glycosides and might have a number of promising prospective applications. AUX, BR, JA, carbon metabolic and flavonoid metabolic pathways all varied between *wsc* and WT and so were selected as important candidates for the generation of pink testae pigmentation (Additional file 20). It is possible that the AUX signal pathway alongside BR and JA synthesis and signaling pathways cooperatively interact to modulate flavonoid synthesis and carbon metabolic pathway-related genes to influence primary metabolism and redirect the accumulation of flavonoids. Three candidate *WSC* gene [*Araip.M7RY3* (*CSN1)*, *Aradu.R8PMF (MYB)* and *Araip.MHR6K (bHLH)*]controls the expression of *FLS* counterparts by regulating hormone signaling and MBW complexes were identified; in turn, this enhance the accumulation of flavonols and negatively controls transcripts of DFR genes resulting in declines in anthocyanins and PA contents (Fig. 7). The SNP in *Araip.MHR6K* (Araip.B06_2335496^[A/G]^) located in the DNA binding domain which might changes its DNA binding ability with the downstream genes (especially the flavonoid synthesis pathway genes) and leads to the white testa phenotype. The SNP could be deployed in genomics-assisted breeding to develop peanut varieties with white testa.

## Conclusions

This study describes a peanut mutant with a white testa that contains higher levels of flavonols and flavone C-glycosides and might have a number of promising prospective applications. Metabolome analysis revealed that isoflavones, flavanones, flavones, flavone C-glycosides, flavonols were all significantly increased while anthocyanins and proanthocyanidins contents markedly decreased. AUX, BR, JA, carbon metabolic and flavonoid metabolic pathways varied between *wsc* and WT and so were selected as important candidates for the generation of white testa pigmentation. It is possible that the AUX signal pathway alongside BR and JA synthesis and signaling pathways coordinate their interaction with modulate flavonoid synthesis and carbon metabolic pathway-related genes to influence primary metabolism and redirect the accumulation of flavonoids. The competition between FLS and DFR modulated by hormone synthesis and signaling as well as the MBW complex might be the key molecular controlling node regulating the white testa phenotype. Combining tissue expression patterns, genetic analyses, and the annotation of common DEGs for these three stages revealed that three testa specific expressed candidate genes, *Araip.M7RY3*, *Aradu.R8PMF* and *Araip.MHR6K* were likely responsible for the white testa phenotype*.* These data corroborate the presence of an interactive relationship between the factors responsible for white testa in *wsc*.

## Methods

### Plant materials and treatments

The *wsc* mutant utilized in this study was isolated from a gradient ^60^Co (100 Gy, 40 Gy.h^− 1^) mutated population (3446 lines) originated by Dr. Liyun Wan from a high yield, high oil content cultivar Zhonghua 16 Cultivated by the Oil Crops Research Institute, Chinese Academy of Agricultural Sciences (OCRI-CAAS). All plants were planted within the experimental plot at OCRI-CAAS in Wuhan, China. Wild type (WT) and *wsc* (M7 generation) individuals were planted in parallel and seed coat samples were collected in 2017 at 20 days after flowering (20DAF), 40DAF, and 60DAF from six different plants. Three biological replicates were performed in each case and the testae separated from each sample seed were sliced. WT and *wsc* testae samples were then rapidly frozen in liquid nitrogen and stored at − 80 °C. Total RNA was isolated from seeds using a RNA extraction kit Qiagen RNeasy Plant Mini kit (QIAGEN Inc.121 Valencia, CA) following the manufacturer’s protocol.

### Tissue preparation and light microscopy observations

Tissue preparation was carried out as described before according to Wan et al. [23]. Sections of the tissue were stained with TBO and safranin O/fast green to reveal polyphenols and suberin, respectively [66]; these were then observed using a Nikon ECLIPSE TI-SR microscope (Nikon Instruments, Japan) after staining and drying.

### Metabolomics and **fatty acid contents**

For testae flavonoids-metabolomics, testae lipid-metabolomics, and embryo untarget-metabolomics, freeze-dried coats were extracted with differentially buffer according to the instructions of METWARE (Wuhan, China) and BGI (Wuhan, China). Sample extracts were analyzed using an LC-ESI-MS/MS system (HPLC, Shim-pack UFLC SHIMADZU CBM30A system; MS, Applied Biosystems 6500 Q TRAP; MS, API 6500 Q TRAP), and quantified as previously described [67]. The metabolite contents were scaled using Z-score of peak area (mean value of three biological replications) in the heatmap.

Extraction and analysis of seed fatty acids was performed as described previously [68].

### RNA sequencing, **data processing, and gene annotation**

Seed coats of WT and *wsc* were harvested at DAF20, DAF40, and DAF60 in 2017 according to their developmental stage and color; these samples were then subject to RNA-seq using an Illumina HiSeq platform at the BGI (Wuhan, China). A 3 μg sample of total RNA from each sample was used to enrich messenger RNA and construct complementary DNA libraries. Thus, use of the internal software SOAPnuke to filter reads yielded a total of 6.66 Gb of clean bases on average for each repeat. HISAT was used for the mapping step on the basis of a peanut synthetic tetraploid reference genome containing *A. duranensis* and *A. ipaensis* [69](https://peautbase.org/). The sequence data have been uploaded in the SRA database of National Center for Biotechnology Information under the accession number of PRJNA497474. A total of 61,903 expressed genes were detected including a total of 7336 predicted as new. We merged a series of novel coding transcripts with reference ones to generate a complete reference set subsequent to the detection of novel transcripts and then mapped clean reads onto this using the software Bowtie2 [70], RSEM was used to calculate gene expression levels for each sample [71]. DEGseq was applied to identify differentially expression genes between samples via algorithms, and resultant *P* values were adjusted using a Benjamini and Hochberg’s correction to control for FDR. Genes determined via DESeq as having an adjusted P value < 0.05 were defined as DEGs. Gene annotation was performed using the software Blast2GO. Functional interpretation of these DEGs was further completed by assigning them to metabolic pathways using KEGG annotation. The WGCNA R package was used to do the coexpression anlysis as described in previous publications [72, 73]. The gene expression was scaled using Z-score of FPKM (mean value of three biological replications) in the heatmap.

### qRT-PCR analysis

Reverse transcriptions and qRT-PCR were carried out as described before according to Wan et al. [23].

### Statistical analysis

The statistical significance was calculated by Student’s *t*-test. The gene expression values of FPKM were scaled to Z-score to draw the heatmap of transcriptome and metabolome. Each experiment was repeated three or six times.

## Supplementary information

**Additional file 1.** Flavonoids detected in peanut testae.

**Additional file 2. **Testae lipid metabolome data between *wsc* and WT.

**Additional file 3. **Embryo metabolome data between *wsc* and WT.

**Additional file 4. **Cotyledon metabolism and fatty acid contents in *wsc* and WT. (A) Enriched KEGG pathways of cotyledon metabolism in *wsc* and WT. (B) Fatty acid contents in *wsc* and WT.

**Additional file 5. **DEGs identified between *wsc* and WT. (XLS 8402 kb)

**Additional file 6. **Gene ontoloty enrichment of the DEGs between *wsc* and WT.

**Additional file 7. **DEGs KEGG pathways between *wsc* and WT.

**Additional file 8.** Gene-specific primers used in qRT-PCR analysis.

**Additional file 9. **qRT-PCR validation of DEGs between *wsc* and WT. (A) Transcript levels of 17 genes with average FPKM value ≥2.5. The y-axis showed relative gene expression levels analyzed by qRT-PCR and RNA-Seq. WT qRT-PCR (rose columns) and *wsc* qRT-PCR (white columns) corresponding to qRT-PCR expression data. WT RNA-Seq (rose lines) and *wsc* RNA-Seq (gray lines) refer to RNA-seq data. The data presented here are mean values from three repetitions. Error bars represent standard error (SE) (*n* = 3). (B) Comparison of gene expression ratios from qRT-PCR and RNA-Seq data. RNA-Seq log2 values of the expression ratio (y-axis) are plotted against the three different developmental stages (x-axis). The gene expression was scaled using Z-score of FPKM (mean value of three biological replications) in the heatmap.

**Additional file 10. **Comparison of glycolysis and citrate cycles between *wsc* and WT. (A) Comparison of glycolysis between *wsc* and WT; (1) hexokinase; (2) glucosephosphate isomerase; (3) phosphofructokinase; (4) aldolase; (5) triose phosphofructokinase; (6) glyceraldehyde phosphate dehydrogenase; (7) phosphoglycerate kinase; (8) phosphoglyceromutase; (9) enolase; (10) pyruvate kinase; (11) non-enzymatic reaction; (12) lactate dehydrogenase; (13) pyruvate decarboxylase; (14) alcohol dehydrogenase. (B) Comparison of the citrate cycle between *wsc* and WT; (pre), pyruvate dehydrogenase complex; (1) citrate synthase; (2) aconitase; (3) aconitase; (4) isocitrate dehydrogenase; (5) α-ketoglutarate dehydrogenase; (6) succinyl CoA synthase; (7) succinate dehydrogenase; (8) funarase; (9) malate dehydrogenase. Gene expression was scaled using Z-scores of FPKM for mean valued of three biological replicates in heatmaps.

**Additional file 11.** FPKM values for DEGs in glycolysis and the TCA cycle. (A) Expression of hexokinase genes. (B) Expression of pyruvate kinases genes. (C) Expression of phosphofructokinase genes. (D) Expression of pyruvate dehydrogenase component genes. (E) Expression of aconitate hydratase genes. (F) Expression of citrate synthase genes. (G) Expression of isocitrate dehydrogenase genes. (H) Expression of succinate dehydrogenase genes. (I) Expression of malate dehydrogenase genes.

**Additional file 12. **Expression profiles of sucrose synthesis and transport genes in *wsc* and WT. SS, sucrose synthase; SUT, sucrose transport protein; STP; sugar transporter protein; ERD, early response to dehydration transporters; SWEET, Sugars Will Eventually be Exported Transporters.

**Additional file 13. **The expression of phytohormone-synthesis pathway genes in *wsc* and WT. (A) Heatmap and FPKM values of different expressed BR synthesis pathway genes between *wsc* and WT. (B) Heatmap and FPKM values of the differently expressed GA synthesis pathway genes between *wsc* and WT. (C) Heatmap and FPKM values of differently expressed JA synthesis pathway genes between *wsc* and WT. BR6OX1, brassinosteroid-6-oxidase 1; BAS1, PHYB-4 ACTIVATION-TAGGED SUPPRESSOR 1; KAO, ent-kaurenoic acid hydroxylase; GA2ox, gibberellin 2-oxidase; GA3ox, gibberellin 3-beta-dioxygenase; PLA1, phospholipase A1; AOC, allene oxide cyclase; OPR, 12-oxophytodienoic acid reductase; MFP2: multifunctional protein 2. The gene expression was scaled using Z-score of FPKM (mean value of three biological replications) in the heatmap.

**Additional file 14. **The expression of mutipl-phytohormones signaling pathway genes in *wsc* and WT. (A) Heatmap and FPKM values for differently expressed ABA signaling pathway genes between *wsc* and WT. (B) Heatmap and FPKM values of differently expressed auxin synthesis pathway genes between *wsc* and WT. (C) Heatmap and FPKM values of differently expressed BR signaling pathway genes between *wsc* and WT. (D) Heatmap and FPKM values of differently expressed CTK signaling pathway genes between *wsc* and WT. (E) Heatmap and FPKM values of different expressed GA signaling pathway genes between *wsc* and WT. (F) Heatmap and FPKM values of differently expressed JA signaling pathway genes between *wsc* and WT. (G) Heatmap and FPKM values of differently expressed SA signaling pathway genes between *wsc* and WT. The gene expression was scaled using Z-score of FPKM (mean value of three biological replications) in the heatmap.

**Additional file 15. **Differentially expressed MYB, bHLH, and WD40 factors between *wsc* and WT. (A) Heatmap and expression levels of differentially expressed MYB transcription factors between *wsc* and WT. (B) Heatmap and expression levels of differentially expressed bHLH factors between *wsc* and WT. (C) Heatmap and expression levels of differentially expressed WD40 factors between *wsc* and WT. The gene expression was scaled using Z-score of FPKM (mean value of three biological replications) in the heatmap.

**Additional file 16.** Expression and annotation of common DEGs at the three developmental stages assessed in this study.

**Additional file 17. **The growing observation of *wsc* and WT.

**Additional file 18.** Expression and annotation of stitching genes between seed specified and common DEGs at the three developmental stages assessed in this study.

**Additional file 19. **Sequence alignment of *Araip.MHR6K* between *wsc* and WT.

**Additional file 20.** WSC losses affect multiple primary and secondly metabolism pathways. Mutant of WSC leads to a coordinated increase in transcript and metabolic levels of carbon metabolism pathways and hormone synthesis and signaling pathways while the flavonoids metabolism pathway was redirected. Up- and down-regulated genes and metabolites are shown in red and green, respectively.

## Data Availability

Transcriptome sequencing data are available in the SRA database of National Center for Biotechnology Information under the accession number of PRJNA497474 (https://www.ncbi.nlm.nih.gov/bioproject/PRJNA497474).

## Abbreviations

ABA: Abscisic acid
ANR: Anthocyanidin reductase
AUX: Auxin
BR: Brassinosteroid
CHI: Chalcone isomerase
CHS: Chalcone synthase
CTK: Cytokinin
DAF: Days after flowering
DEG: Differentially expressed gene
DFR: Dihydroflavonol 4-reductase
EBGs: Early biosynthetic genes
F3H: Flavanone 3-hydroxylase
F3’H: Flavonoid 3′-hydroxylase
F3’5’H: Flavonoid 3′,5′-hydroxylase
FDR: False discovery rate
FLS: Flavonol synthase
FPKM: Fragments per Kilobase Million
GA: Gibberellin
HISAT: Hierarchical Indexing for Spliced Alignment of Transcripts
JA: Jasmonic acid
KEGG: Kyoto Encyclopedia of Genes and Genomes
LAR: Leucoanthocyanidin reductase
LDOX: Leucoanthocyanidin dioxygenase
LBGs: Late biosynthetic genes
MBW: MYB-bHLH-WD40
MeJA: Methyl jasmonate
MTs: Methyltransferases
PA: Proanthocyanidin
POD: Peroxidase
PPOD: Polyphenol oxidase
qRT-PCR: Quantitative reverse-transcription PCR
SA: Salicylic acid
SUTs: H^+^/sucrose symporters
TBO: Toluidine blue O
TCA: Tricarboxylic acid
TSTs: The H^+^/sucrose antiporters located on tonoplast
UFGT: UDP-glucose:flavonoid 3-O-glucosyl transferase
*wsc*: White seed coat peanut mutant
WT: Wild Type
