# Supplementary material for: Transcriptome and metabolome reveal redirection of flavonoids in a white testa peanut mutant
Source: BMC Plant Biol. 2020 Apr 15;20:161. doi: 10.1186/s12870-020-02383-7 (PMC7161308; doi:10.1186/s12870-020-02383-7)
Supplement: Supplementary file 4 — Additional file 4. Cotyledon metabolism and fatty acid contents in wsc and WT. (A) Enriched KEGG pathways of cotyledon metabolism in wsc and WT. (B) Fatty acid contents in wsc and WT. [file 12870_2020_2383_MOESM4_ESM.ppt]

## Slide 1
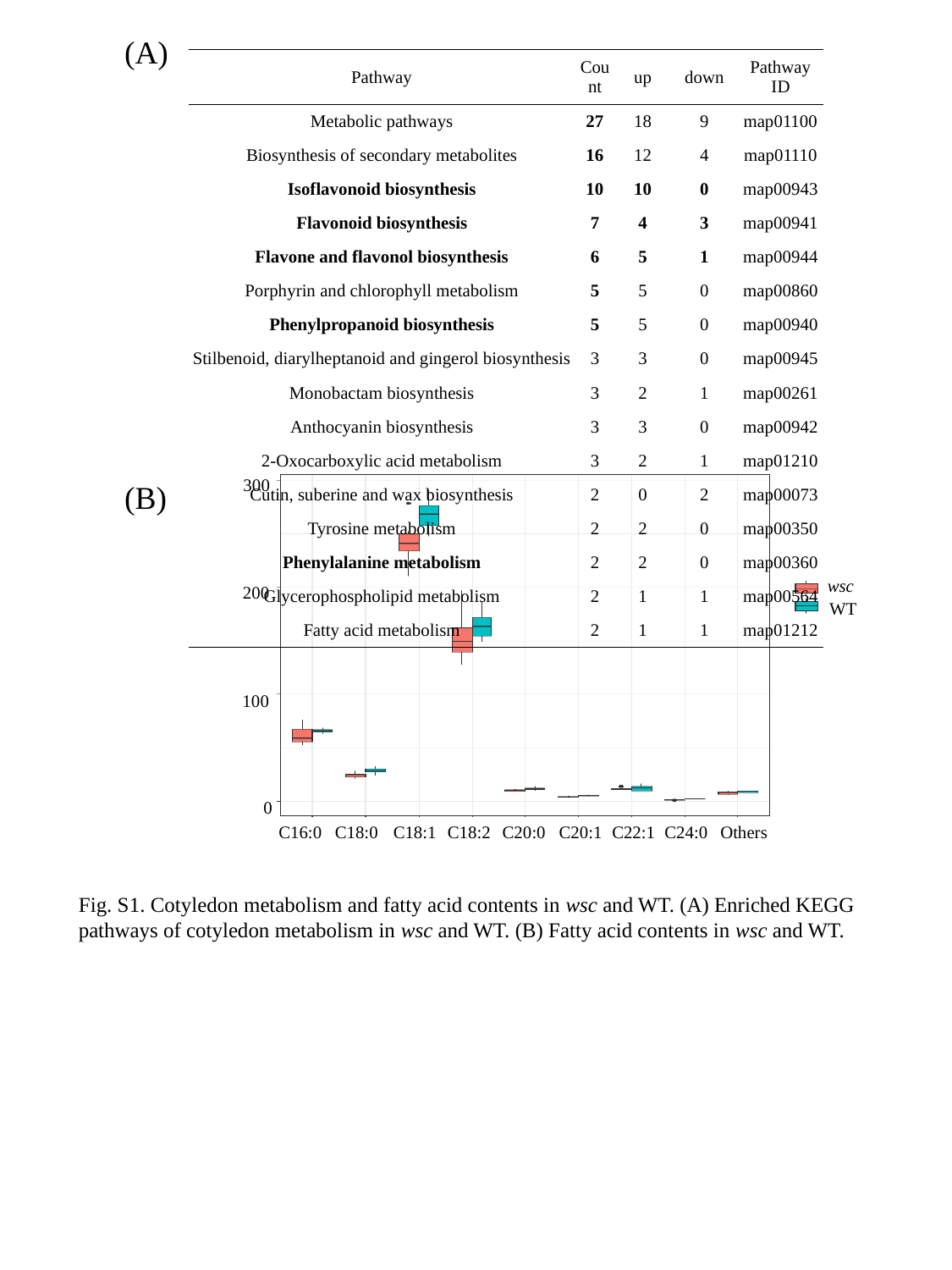

(A)
| Pathway | Count | up | down | Pathway ID |
| --- | --- | --- | --- | --- |
| Metabolic pathways | 27 | 18 | 9 | map01100 |
| Biosynthesis of secondary metabolites | 16 | 12 | 4 | map01110 |
| Isoflavonoid biosynthesis | 10 | 10 | 0 | map00943 |
| Flavonoid biosynthesis | 7 | 4 | 3 | map00941 |
| Flavone and flavonol biosynthesis | 6 | 5 | 1 | map00944 |
| Porphyrin and chlorophyll metabolism | 5 | 5 | 0 | map00860 |
| Phenylpropanoid biosynthesis | 5 | 5 | 0 | map00940 |
| Stilbenoid, diarylheptanoid and gingerol biosynthesis | 3 | 3 | 0 | map00945 |
| Monobactam biosynthesis | 3 | 2 | 1 | map00261 |
| Anthocyanin biosynthesis | 3 | 3 | 0 | map00942 |
| 2-Oxocarboxylic acid metabolism | 3 | 2 | 1 | map01210 |
| Cutin, suberine and wax biosynthesis | 2 | 0 | 2 | map00073 |
| Tyrosine metabolism | 2 | 2 | 0 | map00350 |
| Phenylalanine metabolism | 2 | 2 | 0 | map00360 |
| Glycerophospholipid metabolism | 2 | 1 | 1 | map00564 |
| Fatty acid metabolism | 2 | 1 | 1 | map01212 |
300
wsc
200
WT
100
0
C16:0
C18:0
C18:1
C18:2
C20:0
C20:1
C22:1
C24:0
Others
(B)
Fig. S1. Cotyledon metabolism and fatty acid contents in wsc and WT. (A) Enriched KEGG pathways of cotyledon metabolism in wsc and WT. (B) Fatty acid contents in wsc and WT.
