# Supplementary material for: Transcriptome and metabolome reveal redirection of flavonoids in a white testa peanut mutant
Source: BMC Plant Biol. 2020 Apr 15;20:161. doi: 10.1186/s12870-020-02383-7 (PMC7161308; doi:10.1186/s12870-020-02383-7)
Supplement: Supplementary file 9 — Additional file 9. qRT-PCR validation of DEGs between wsc and WT. (A) Transcript levels of 17 genes with average FPKM value ≥2.5. The y-axis showed relative gene expression levels analyzed by qRT-PCR and RNA-Seq. WT qRT-PCR (rose columns) and wsc qRT-PCR (white columns) corresponding to qRT-PCR expression data. WT RNA-Seq (rose lines) and wsc RNA-Seq (gray lines) refer to RNA-seq data. The data presented here are mean values from three repetitions. Error bars represent standard error (SE) (n = 3). (B) Comparison of gene expression ratios from qRT-PCR and RNA-Seq data. RNA-Seq log2 values of the expression ratio (y-axis) are plotted against the three different developmental stages (x-axis). The gene expression was scaled using Z-score of FPKM (mean value of three biological replications) in the heatmap. [file 12870_2020_2383_MOESM9_ESM.ppt]

## Slide 1
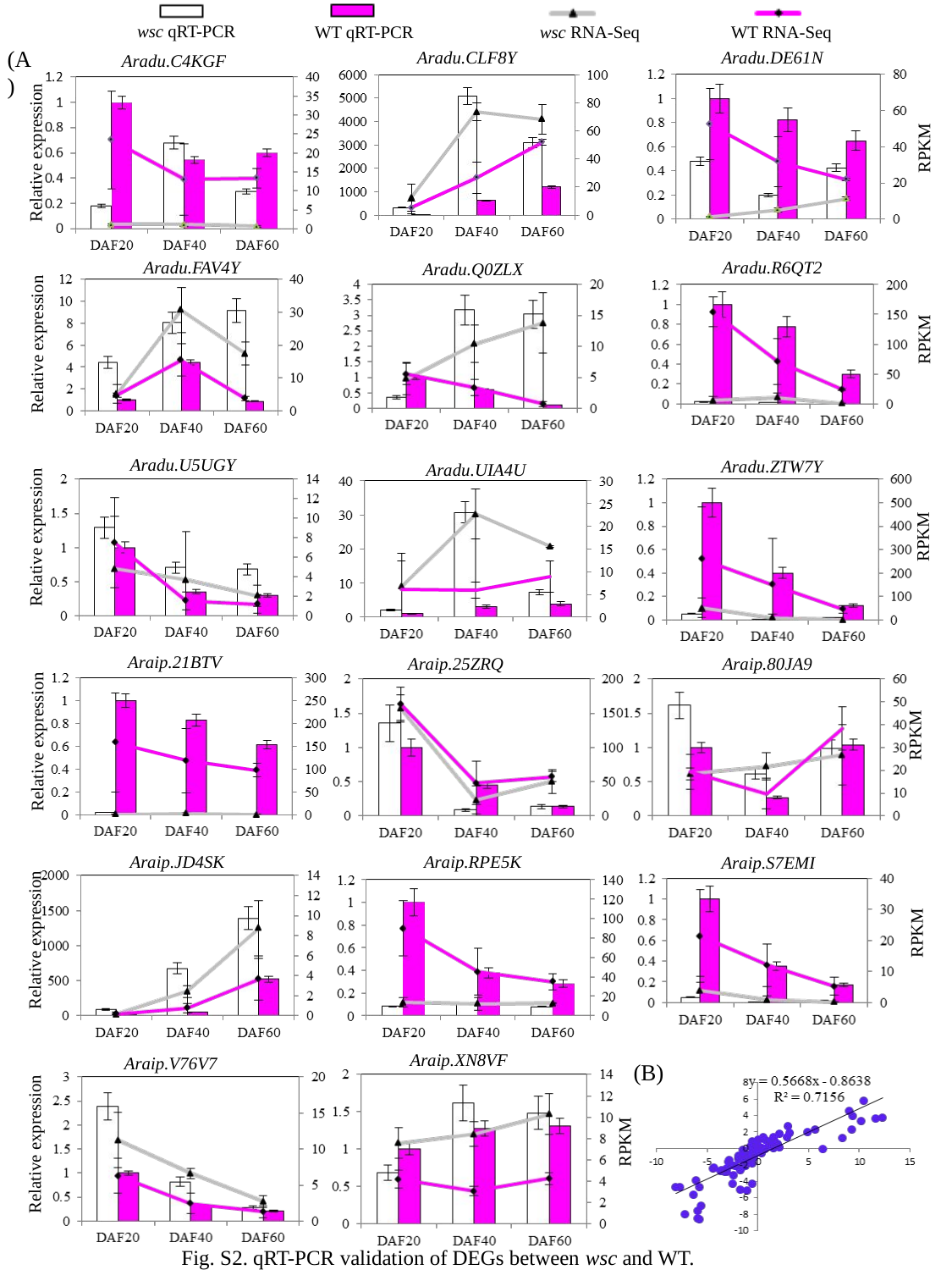

wsc qRT-PCR
wsc RNA-Seq
WT qRT-PCR
WT RNA-Seq
Relative expression
(A)
Aradu.CLF8Y
Aradu.DE61N
Aradu.C4KGF
RPKM
Relative expression
Aradu.FAV4Y
Aradu.R6QT2
Aradu.Q0ZLX
RPKM
Relative expression
Aradu.U5UGY
Aradu.ZTW7Y
RPKM
Aradu.UIA4U
Relative expression
Araip.21BTV
Araip.80JA9
Araip.25ZRQ
RPKM
Relative expression
Araip.RPE5K
Araip.JD4SK
Araip.S7EMI
RPKM
Relative expression
Araip.XN8VF
Araip.V76V7
(B)
RPKM
Fig. S2. qRT-PCR validation of DEGs between wsc and WT.
