# Supplementary material for: Transcriptome and metabolome reveal redirection of flavonoids in a white testa peanut mutant
Source: BMC Plant Biol. 2020 Apr 15;20:161. doi: 10.1186/s12870-020-02383-7 (PMC7161308; doi:10.1186/s12870-020-02383-7)
Supplement: Supplementary file 10 — Additional file 10. Comparison of glycolysis and citrate cycles between wsc and WT. (A) Comparison of glycolysis between wsc and WT; (1) hexokinase; (2) glucosephosphate isomerase; (3) phosphofructokinase; (4) aldolase; (5) triose phosphofructokinase; (6) glyceraldehyde phosphate dehydrogenase; (7) phosphoglycerate kinase; (8) phosphoglyceromutase; (9) enolase; (10) pyruvate kinase; (11) non-enzymatic reaction; (12) lactate dehydrogenase; (13) pyruvate decarboxylase; (14) alcohol dehydrogenase. (B) Comparison of the citrate cycle between wsc and WT; (pre), pyruvate dehydrogenase complex; (1) citrate synthase; (2) aconitase; (3) aconitase; (4) isocitrate dehydrogenase; (5) α-ketoglutarate dehydrogenase; (6) succinyl CoA synthase; (7) succinate dehydrogenase; (8) funarase; (9) malate dehydrogenase. Gene expression was scaled using Z-scores of FPKM for mean valued of three biological replicates in heatmaps. [file 12870_2020_2383_MOESM10_ESM.ppt]

## Slide 1
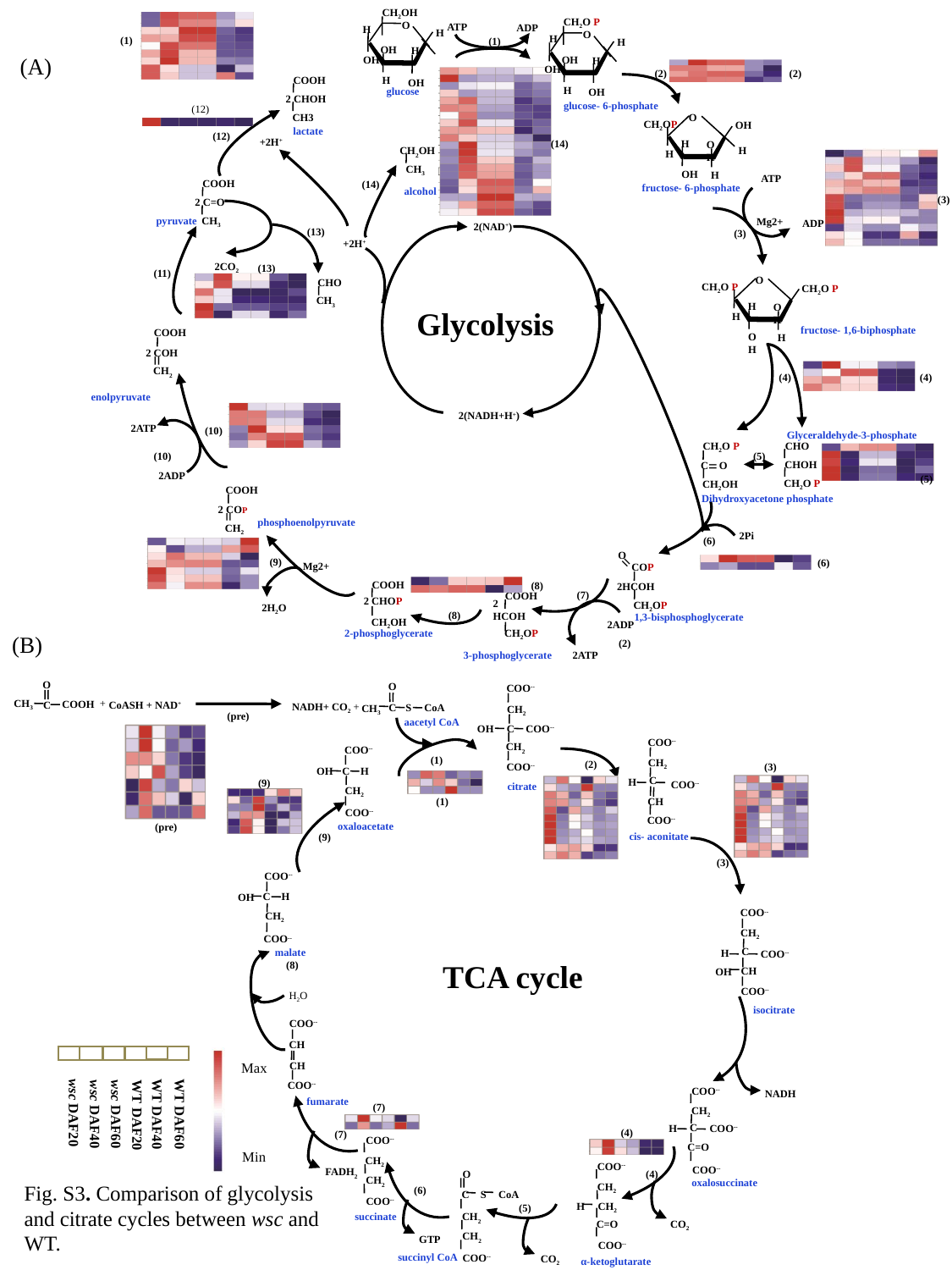

CH2OH
O
H
H
OH
H
OH
H
CH2O P
O
H
H
OH
H
OH
H
ATP
ADP
(1)
(1)
(A)
(2)
(2)
 2 CHOH
CH3
COOH
OH
glucose
OH
glucose- 6-phosphate
(12)
O
CH2OP
OH
H
OH
H
OH
H
H
lactate
(12)
+2H+
(14)
 CH2OH
CH3
ATP
 2 C=O
CH3
COOH
(14)
fructose- 6-phosphate
alcohol
(3)
pyruvate
Mg2+
ADP
2(NAD+)
(13)
(3)
+2H+
2CO2
(13)
(11)
O
CH2O P
CH2O P
H
OH
H
OH
H
 CHO
CH3
Glycolysis
fructose- 1,6-biphosphate
COOH
 2 COH
CH2
=
(4)
(4)
enolpyruvate
2(NADH+H+)
2ATP
(10)
Glyceraldehyde-3-phosphate
CHOH
CH2O P
CH2O P
=
C
CH2OH
O
CHO
(10)
(5)
2ADP
(5)
 2 COP
CH2
COOH
=
Dihydroxyacetone phosphate
phosphoenolpyruvate
2Pi
(6)
O
=
2HCOH
CH2OP
COP
(9)
(6)
Mg2+
 2 CHOP
CH2OH
COOH
(8)
(7)
2 HCOH
CH2OP
COOH
2H2O
(8)
1,3-bisphosphoglycerate
2ADP
2-phosphoglycerate
(B)
(2)
3-phosphoglycerate
2ATP
O
CH3
COOH
C
O
CoA
C
S
CH3
COO--
OH
COO--
COO--
CH2
 C
CH2
+
CoASH + NAD+
NADH+ CO2
+
(pre)
aacetyl CoA
COO--
H
COO--
COO--
CH2
 C
CH
COO--
COO--
C H
 CH2
OH
(1)
(2)
(3)
(9)
citrate
(1)
oxaloacetate
(pre)
cis- aconitate
(9)
(3)
COO--
COO--
C H
 CH2
OH
COO--
COO--
OH
COO--
CH2
 C
CH
H
malate
TCA cycle
(8)
H2O
isocitrate
COO--
COO--
CH
 CH
wsc DAF20
WT DAF40
WT DAF60
wsc DAF40
wsc DAF60
WT DAF20
Max
COO--
COO--
COO--
 C
C=O
CH2
H
NADH
fumarate
(7)
(4)
(7)
COO--
COO--
 CH2
CH2
Min
COO--
COO--
 CH2
C=O
CH2
H
FADH2
O
S
CoA
C
(4)
oxalosuccinate
Fig. S3. Comparison of glycolysis and citrate cycles between wsc and WT.
(6)
(5)
COO--
 CH2
CH2
succinate
CO2
GTP
succinyl CoA
CO2
α-ketoglutarate
