# Supplementary material for: Transcriptome and metabolome reveal redirection of flavonoids in a white testa peanut mutant
Source: BMC Plant Biol. 2020 Apr 15;20:161. doi: 10.1186/s12870-020-02383-7 (PMC7161308; doi:10.1186/s12870-020-02383-7)
Supplement: Supplementary file 11 — Additional file 11. FPKM values for DEGs in glycolysis and the TCA cycle. (A) Expression of hexokinase genes. (B) Expression of pyruvate kinases genes. (C) Expression of phosphofructokinase genes. (D) Expression of pyruvate dehydrogenase component genes. (E) Expression of aconitate hydratase genes. (F) Expression of citrate synthase genes. (G) Expression of isocitrate dehydrogenase genes. (H) Expression of succinate dehydrogenase genes. (I) Expression of malate dehydrogenase genes. [file 12870_2020_2383_MOESM11_ESM.ppt]

## Slide 1
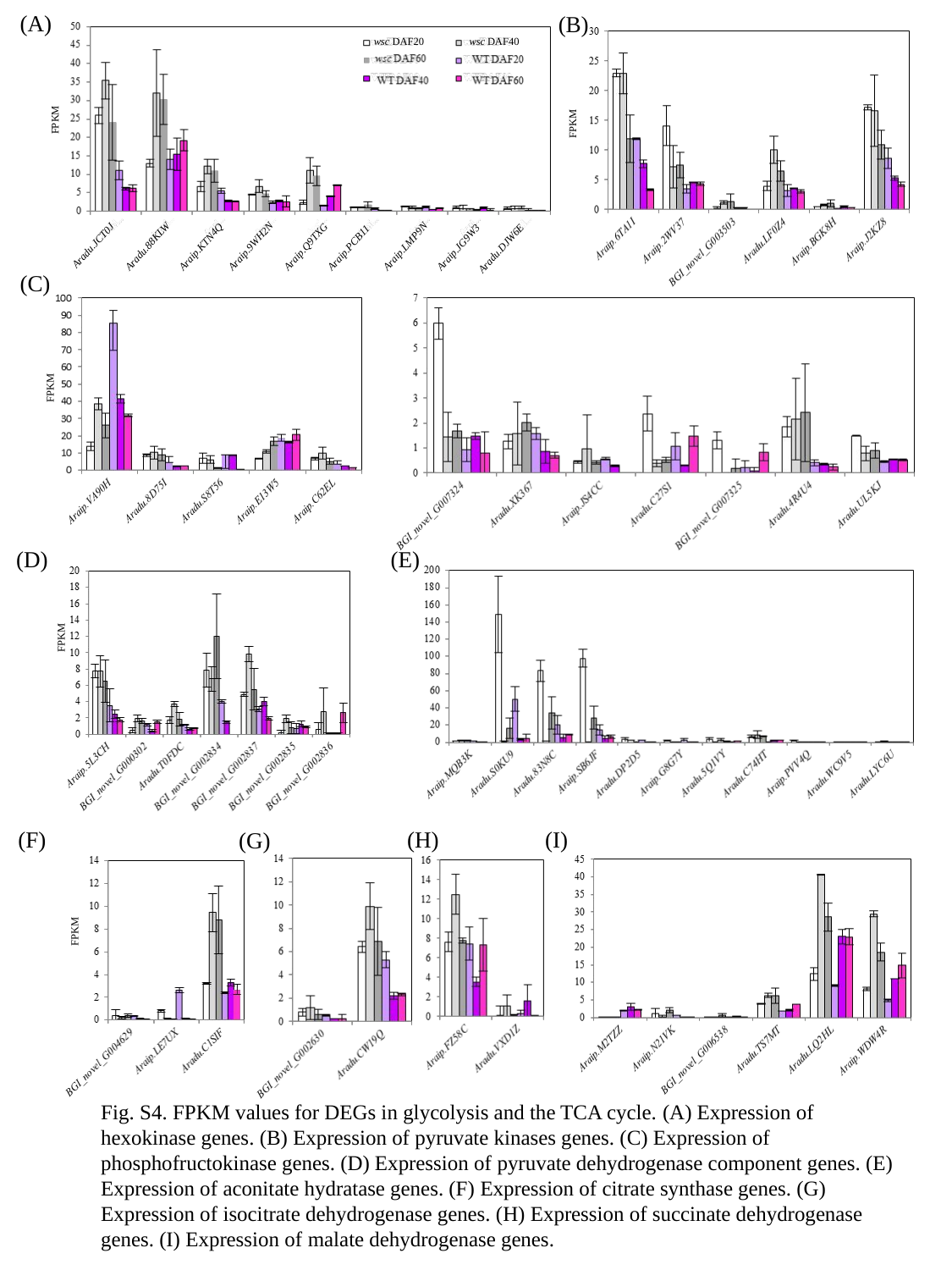

(A)
(B)
wsc DAF20
wsc DAF40
FPKM
FPKM
Aradu.88KLW
Araip.9WH2N
Araip.Q9TXG
Araip.PCB11
Araip.LMP9N
Araip.KTN4Q
Araip.JG9W3
Aradu.DJW6E
Aradu.JCT0J
(C)
FPKM
(D)
(E)
FPKM
(F)
(H)
(I)
(G)
FPKM
Fig. S4. FPKM values for DEGs in glycolysis and the TCA cycle. (A) Expression of hexokinase genes. (B) Expression of pyruvate kinases genes. (C) Expression of phosphofructokinase genes. (D) Expression of pyruvate dehydrogenase component genes. (E) Expression of aconitate hydratase genes. (F) Expression of citrate synthase genes. (G) Expression of isocitrate dehydrogenase genes. (H) Expression of succinate dehydrogenase genes. (I) Expression of malate dehydrogenase genes.
