# Supplementary material for: Transcriptome and metabolome reveal redirection of flavonoids in a white testa peanut mutant
Source: BMC Plant Biol. 2020 Apr 15;20:161. doi: 10.1186/s12870-020-02383-7 (PMC7161308; doi:10.1186/s12870-020-02383-7)
Supplement: Supplementary file 12 — Additional file 12. Expression profiles of sucrose synthesis and transport genes in wsc and WT. SS, sucrose synthase; SUT, sucrose transport protein; STP; sugar transporter protein; ERD, early response to dehydration transporters; SWEET, Sugars Will Eventually be Exported Transporters. [file 12870_2020_2383_MOESM12_ESM.ppt]

## Slide 1
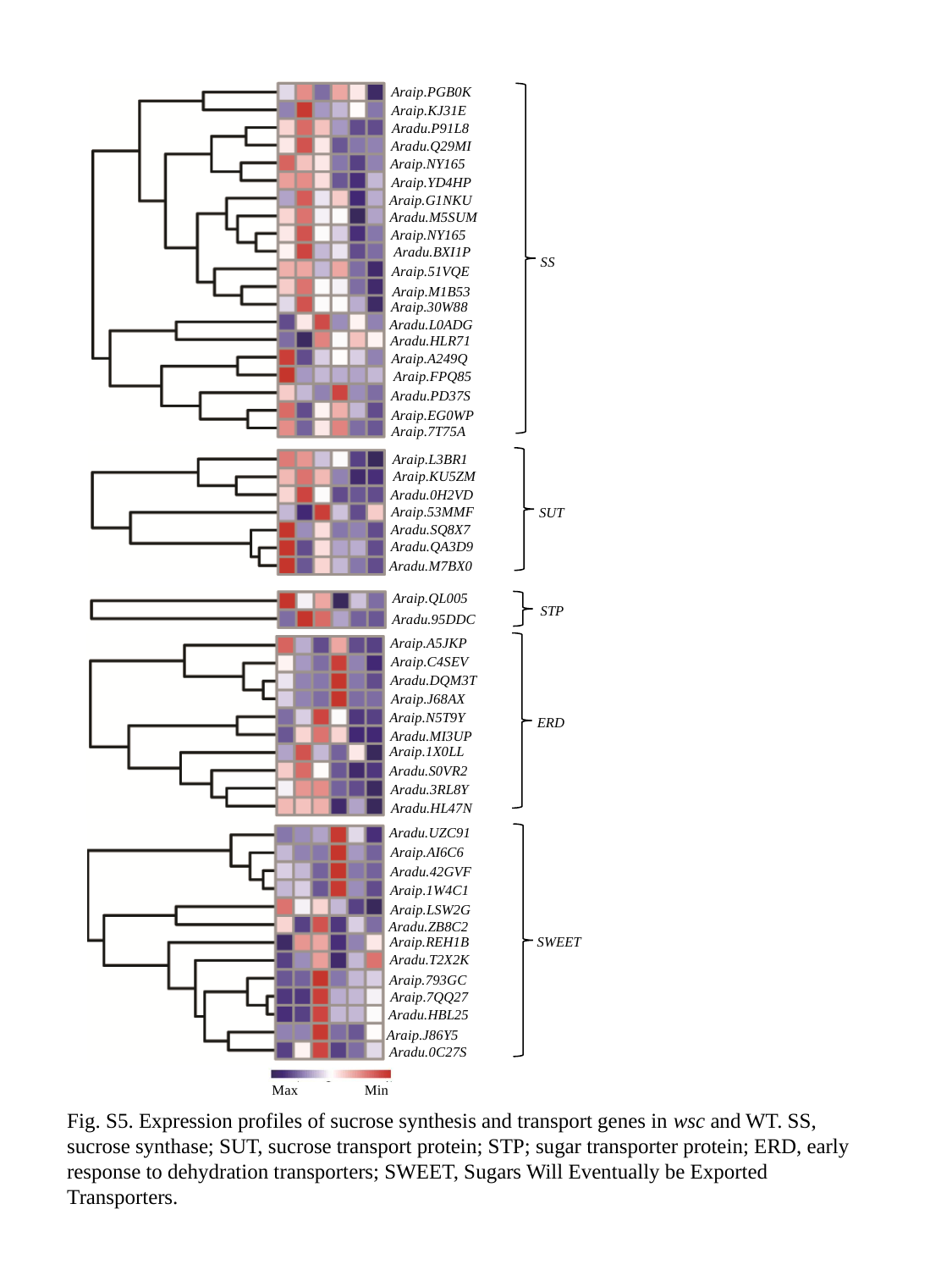

Araip.PGB0K
Araip.KJ31E
Aradu.P91L8
Aradu.Q29MI
Araip.NY165
Araip.YD4HP
Araip.G1NKU
Aradu.M5SUM
Araip.NY165
Aradu.BXI1P
SS
Araip.51VQE
Araip.M1B53
Araip.30W88
Aradu.L0ADG
Aradu.HLR71
Araip.A249Q
Araip.FPQ85
Aradu.PD37S
Araip.EG0WP
Araip.7T75A
Araip.L3BR1
Araip.KU5ZM
Aradu.0H2VD
Araip.53MMF
SUT
Aradu.SQ8X7
Aradu.QA3D9
Aradu.M7BX0
Araip.QL005
STP
Aradu.95DDC
Araip.A5JKP
Araip.C4SEV
Aradu.DQM3T
Araip.J68AX
Araip.N5T9Y
ERD
Aradu.MI3UP
Araip.1X0LL
Aradu.S0VR2
Aradu.3RL8Y
Aradu.HL47N
Aradu.UZC91
Araip.AI6C6
Aradu.42GVF
Araip.1W4C1
Araip.LSW2G
Aradu.ZB8C2
Araip.REH1B
SWEET
Aradu.T2X2K
Araip.793GC
Araip.7QQ27
Aradu.HBL25
Araip.J86Y5
Aradu.0C27S
Max
Min
Fig. S5. Expression profiles of sucrose synthesis and transport genes in wsc and WT. SS, sucrose synthase; SUT, sucrose transport protein; STP; sugar transporter protein; ERD, early response to dehydration transporters; SWEET, Sugars Will Eventually be Exported Transporters.
