# Supplementary material for: Transcriptome and metabolome reveal redirection of flavonoids in a white testa peanut mutant
Source: BMC Plant Biol. 2020 Apr 15;20:161. doi: 10.1186/s12870-020-02383-7 (PMC7161308; doi:10.1186/s12870-020-02383-7)
Supplement: Supplementary file 13 — Additional file 13. The expression of phytohormone-synthesis pathway genes in wsc and WT. (A) Heatmap and FPKM values of different expressed BR synthesis pathway genes between wsc and WT. (B) Heatmap and FPKM values of the differently expressed GA synthesis pathway genes between wsc and WT. (C) Heatmap and FPKM values of differently expressed JA synthesis pathway genes between wsc and WT. BR6OX1, brassinosteroid-6-oxidase 1; BAS1, PHYB-4 ACTIVATION-TAGGED SUPPRESSOR 1; KAO, ent-kaurenoic acid hydroxylase; GA2ox, gibberellin 2-oxidase; GA3ox, gibberellin 3-beta-dioxygenase; PLA1, phospholipase A1; AOC, allene oxide cyclase; OPR, 12-oxophytodienoic acid reductase; MFP2: multifunctional protein 2. The gene expression was scaled using Z-score of FPKM (mean value of three biological replications) in the heatmap. [file 12870_2020_2383_MOESM13_ESM.ppt]

## Slide 1
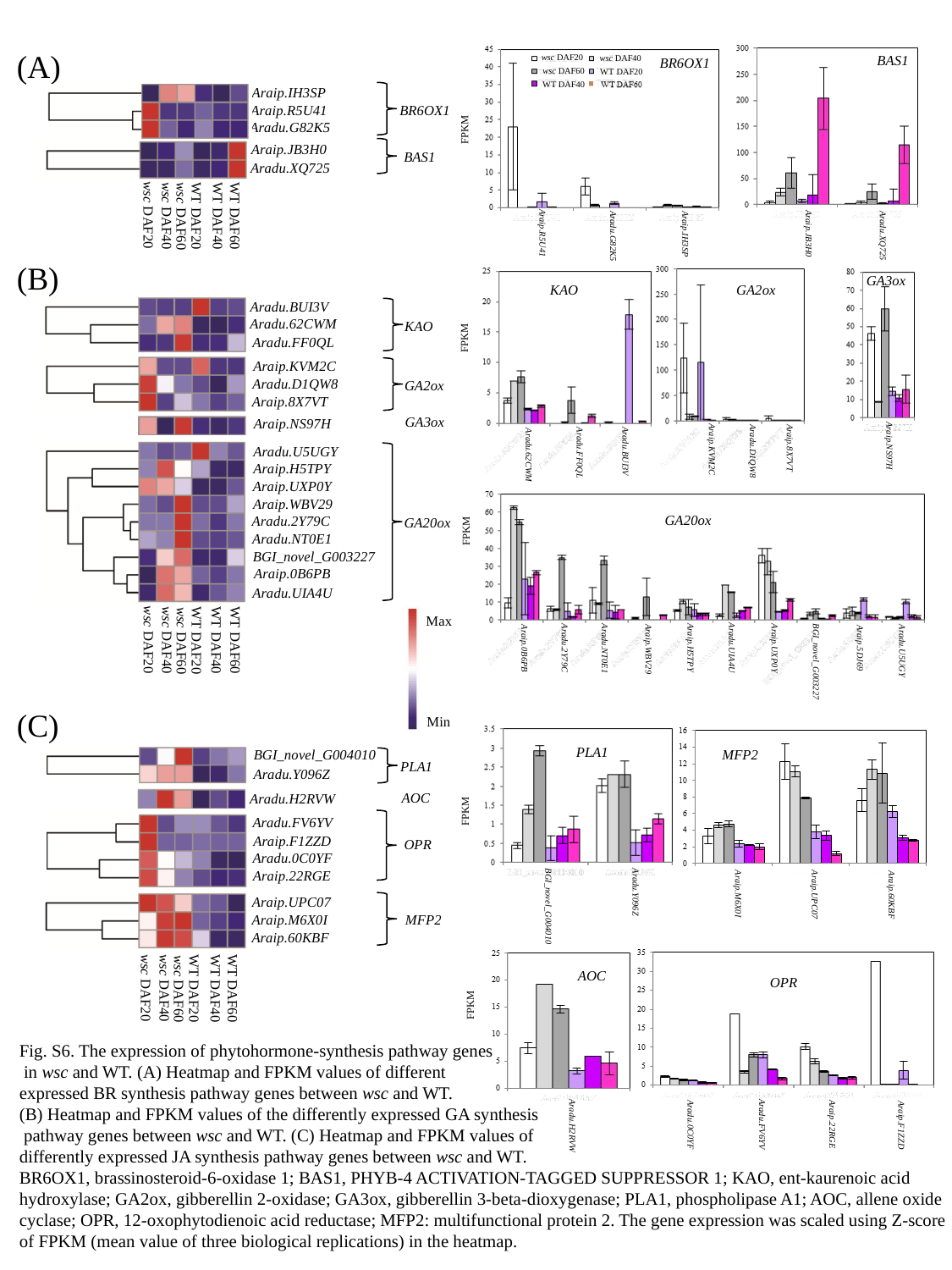

(A)
 BAS1
wsc DAF20
BR6OX1
wsc DAF40
wsc DAF60
WT DAF20
WT DAF40
Araip.IH3SP
FPKM
BR6OX1
Araip.R5U41
Aradu.G82K5
Araip.JB3H0
 BAS1
Aradu.XQ725
wsc DAF20
WT DAF40
WT DAF60
wsc DAF40
WT DAF20
wsc DAF60
Araip.R5U41
Araip.IH3SP
Aradu.G82K5
Araip.JB3H0
Aradu.XQ725
(B)
GA3ox
KAO
GA2ox
FPKM
Aradu.BUI3V
Aradu.62CWM
KAO
Aradu.FF0QL
Araip.KVM2C
Aradu.D1QW8
GA2ox
Araip.8X7VT
GA3ox
Araip.NS97H
Aradu.U5UGY
Araip.NS97H
Araip.KVM2C
Araip.8X7VT
Aradu.D1QW8
Aradu.BUI3V
Aradu.FF0QL
Aradu.62CWM
Araip.H5TPY
Araip.UXP0Y
Araip.WBV29
FPKM
GA20ox
Aradu.2Y79C
GA20ox
Aradu.NT0E1
BGI_novel_G003227
Araip.0B6PB
Aradu.UIA4U
wsc DAF20
WT DAF40
WT DAF60
wsc DAF40
WT DAF20
wsc DAF60
Max
Min
Aradu.UIA4U
Araip.H5TPY
Araip.UXP0Y
Aradu.NT0E1
Aradu.2Y79C
Araip.WBV29
Araip.0B6PB
Aradu.U5UGY
Araip.5DJ69
BGI_novel_G003227
(C)
PLA1
MFP2
BGI_novel_G004010
PLA1
Aradu.Y096Z
FPKM
AOC
Aradu.H2RVW
Aradu.FV6YV
Araip.F1ZZD
OPR
Aradu.0C0YF
Araip.22RGE
Araip.UPC07
Aradu.Y096Z
Araip.M6X0I
Araip.UPC07
Araip.60KBF
BGI_novel_G004010
MFP2
Araip.M6X0I
Araip.60KBF
wsc DAF20
WT DAF40
WT DAF60
wsc DAF40
WT DAF20
wsc DAF60
FPKM
AOC
OPR
Fig. S6. The expression of phytohormone-synthesis pathway genes
 in wsc and WT. (A) Heatmap and FPKM values of different
expressed BR synthesis pathway genes between wsc and WT.
(B) Heatmap and FPKM values of the differently expressed GA synthesis
 pathway genes between wsc and WT. (C) Heatmap and FPKM values of
differently expressed JA synthesis pathway genes between wsc and WT.
BR6OX1, brassinosteroid-6-oxidase 1; BAS1, PHYB-4 ACTIVATION-TAGGED SUPPRESSOR 1; KAO, ent-kaurenoic acid hydroxylase; GA2ox, gibberellin 2-oxidase; GA3ox, gibberellin 3-beta-dioxygenase; PLA1, phospholipase A1; AOC, allene oxide cyclase; OPR, 12-oxophytodienoic acid reductase; MFP2: multifunctional protein 2. The gene expression was scaled using Z-score of FPKM (mean value of three biological replications) in the heatmap.
Aradu.FV6YV
Aradu.H2RVW
Araip.22RGE
Aradu.0C0YF
Araip.F1ZZD
