# Supplementary material for: Transcriptome and metabolome reveal redirection of flavonoids in a white testa peanut mutant
Source: BMC Plant Biol. 2020 Apr 15;20:161. doi: 10.1186/s12870-020-02383-7 (PMC7161308; doi:10.1186/s12870-020-02383-7)
Supplement: Supplementary file 14 — Additional file 14. The expression of mutipl-phytohormones signaling pathway genes in wsc and WT. (A) Heatmap and FPKM values for differently expressed ABA signaling pathway genes between wsc and WT. (B) Heatmap and FPKM values of differently expressed auxin synthesis pathway genes between wsc and WT. (C) Heatmap and FPKM values of differently expressed BR signaling pathway genes between wsc and WT. (D) Heatmap and FPKM values of differently expressed CTK signaling pathway genes between wsc and WT. (E) Heatmap and FPKM values of different expressed GA signaling pathway genes between wsc and WT. (F) Heatmap and FPKM values of differently expressed JA signaling pathway genes between wsc and WT. (G) Heatmap and FPKM values of differently expressed SA signaling pathway genes between wsc and WT. The gene expression was scaled using Z-score of FPKM (mean value of three biological replications) in the heatmap. [file 12870_2020_2383_MOESM14_ESM.ppt]

## Slide 1
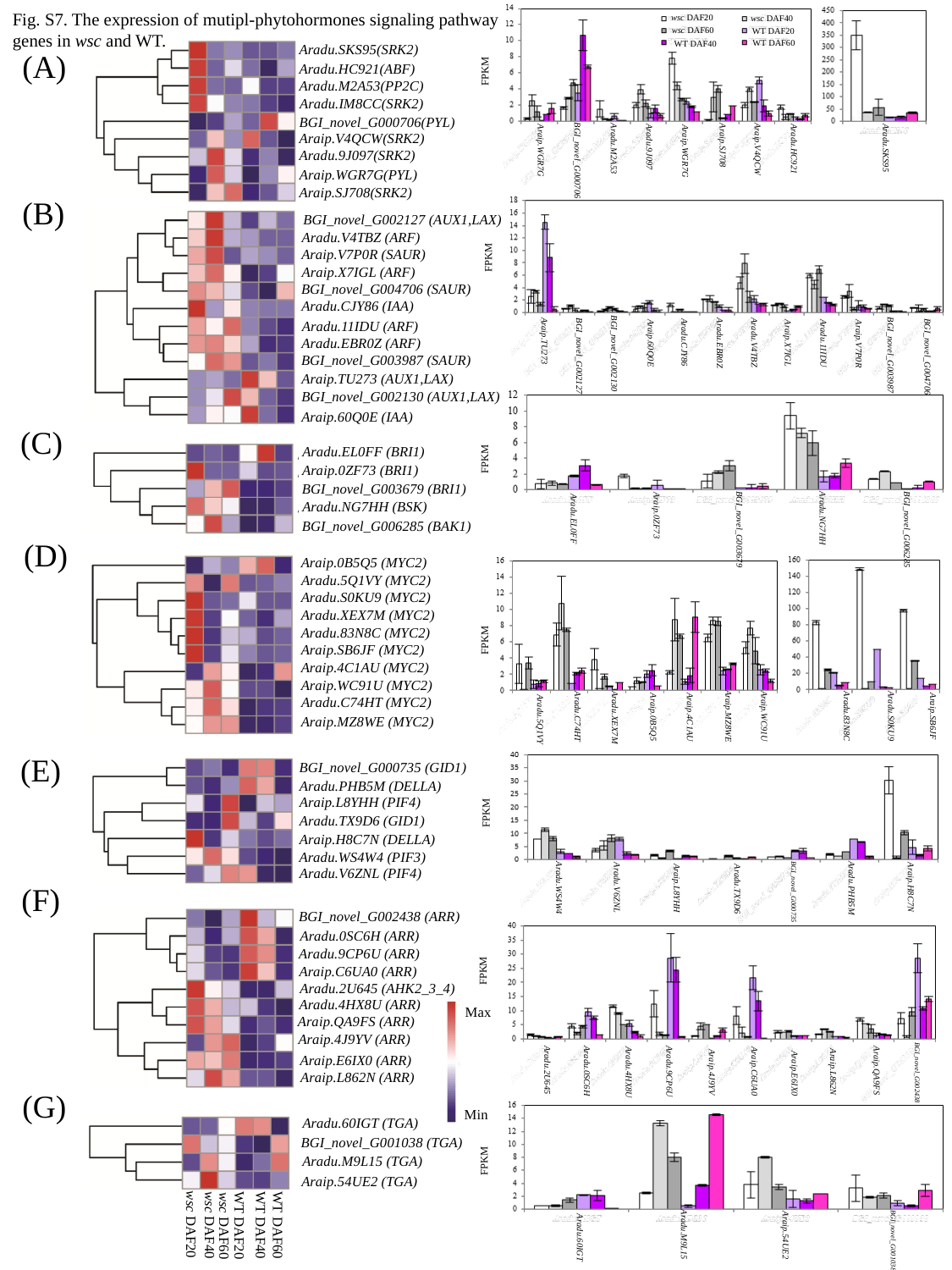

FPKM
Araip.SJ708
Aradu.9J097
Aradu.SKS95
Aradu.M2A53
Aradu.HC921
Araip.V4QCW
Araip.WGR7G
Araip.WGR7G
BGI_novel_G000706
Fig. S7. The expression of mutipl-phytohormones signaling pathway genes in wsc and WT.
wsc DAF20
wsc DAF40
wsc DAF60
WT DAF20
WT DAF60
WT DAF40
Aradu.SKS95(SRK2)
(A)
Aradu.HC921(ABF)
Aradu.M2A53(PP2C)
Aradu.IM8CC(SRK2)
BGI_novel_G000706(PYL)
Araip.V4QCW(SRK2)
Aradu.9J097(SRK2)
FPKM
Araip.TU273
Aradu.CJY86
Aradu.V4TBZ
Araip.60Q0E
Aradu.EBR0Z
Araip.X7IGL
Aradu.11IDU
Araip.V7P0R
BGI_novel_G002130
BGI_novel_G003987
BGI_novel_G002127
BGI_novel_G004706
Araip.WGR7G(PYL)
Araip.SJ708(SRK2)
(B)
BGI_novel_G002127 (AUX1,LAX)
Aradu.V4TBZ (ARF)
Araip.V7P0R (SAUR)
Araip.X7IGL (ARF)
BGI_novel_G004706 (SAUR)
Aradu.CJY86 (IAA)
Aradu.11IDU (ARF)
Aradu.EBR0Z (ARF)
BGI_novel_G003987 (SAUR)
FPKM
Araip.0ZF73
Aradu.NG7HH
Aradu.EL0FF
BGI_novel_G003679
BGI_novel_G006285
Araip.TU273 (AUX1,LAX)
BGI_novel_G002130 (AUX1,LAX)
Araip.60Q0E (IAA)
(C)
Aradu.EL0FF (BRI1)
Araip.0ZF73 (BRI1)
BGI_novel_G003679 (BRI1)
Aradu.NG7HH (BSK)
BGI_novel_G006285 (BAK1)
(D)
FPKM
Aradu.83N8C
Aradu.S0KU9
Araip.SB6JF
Araip.MZ8WE
Araip.WC91U
Aradu.C74HT
Araip.0B5Q5
Araip.4C1AU
Aradu.XEX7M
Aradu.5Q1VY
Araip.0B5Q5 (MYC2)
Aradu.5Q1VY (MYC2)
Aradu.S0KU9 (MYC2)
Aradu.XEX7M (MYC2)
Aradu.83N8C (MYC2)
Araip.SB6JF (MYC2)
Araip.4C1AU (MYC2)
Araip.WC91U (MYC2)
Aradu.C74HT (MYC2)
Araip.MZ8WE (MYC2)
FPKM
Araip.H8C7N
Aradu.V6ZNL
Aradu.PHB5M
Aradu.WS4W4
Araip.L8YHH
Aradu.TX9D6
BGI_novel_G000735
(E)
BGI_novel_G000735 (GID1)
Aradu.PHB5M (DELLA)
Araip.L8YHH (PIF4)
Aradu.TX9D6 (GID1)
Araip.H8C7N (DELLA)
Aradu.WS4W4 (PIF3)
Aradu.V6ZNL (PIF4)
FPKM
Aradu.2U645
Araip.QA9FS
Aradu.0SC6H
Aradu.9CP6U
Araip.C6UA0
Araip.L862N
Araip.4J9YV
Aradu.4HX8U
Araip.E6IX0
BGI_novel_G002438
(F)
BGI_novel_G002438 (ARR)
Aradu.0SC6H (ARR)
Aradu.9CP6U (ARR)
Araip.C6UA0 (ARR)
Aradu.2U645 (AHK2_3_4)
Aradu.4HX8U (ARR)
Araip.QA9FS (ARR)
Araip.4J9YV (ARR)
Araip.E6IX0 (ARR)
Araip.L862N (ARR)
Max
Min
FPKM
Aradu.M9L15
Araip.54UE2
Aradu.60IGT
BGI_novel_G001038
Aradu.60IGT (TGA)
BGI_novel_G001038 (TGA)
Aradu.M9L15 (TGA)
Araip.54UE2 (TGA)
wsc DAF20
WT DAF40
WT DAF60
wsc DAF40
WT DAF20
wsc DAF60
(G)
