# Supplementary material for: Transcriptome and metabolome reveal redirection of flavonoids in a white testa peanut mutant
Source: BMC Plant Biol. 2020 Apr 15;20:161. doi: 10.1186/s12870-020-02383-7 (PMC7161308; doi:10.1186/s12870-020-02383-7)
Supplement: Supplementary file 15 — Additional file 15. Differentially expressed MYB, bHLH, and WD40 factors between wsc and WT. (A) Heatmap and expression levels of differentially expressed MYB transcription factors between wsc and WT. (B) Heatmap and expression levels of differentially expressed bHLH factors between wsc and WT. (C) Heatmap and expression levels of differentially expressed WD40 factors between wsc and WT. The gene expression was scaled using Z-score of FPKM (mean value of three biological replications) in the heatmap. [file 12870_2020_2383_MOESM15_ESM.ppt]

## Slide 1
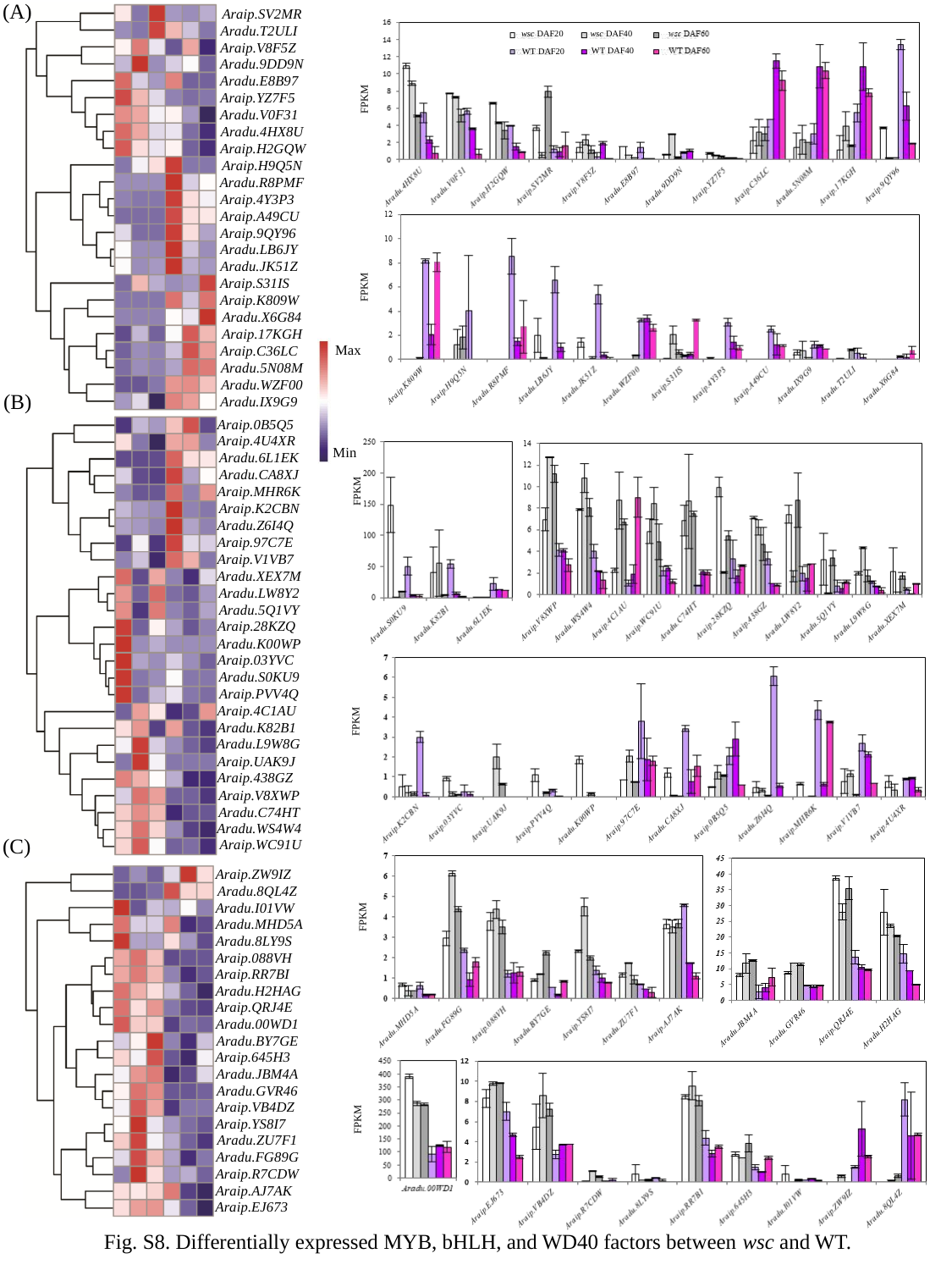

(A)
Araip.SV2MR
Aradu.T2ULI
wsc DAF20
wsc DAF40
Araip.V8F5Z
WT DAF40
WT DAF20
Aradu.9DD9N
FPKM
Aradu.E8B97
Araip.YZ7F5
Aradu.V0F31
Aradu.4HX8U
Araip.H2GQW
Araip.H9Q5N
Aradu.R8PMF
Araip.4Y3P3
Araip.A49CU
Araip.9QY96
Aradu.LB6JY
FPKM
Aradu.JK51Z
Araip.S31IS
Araip.K809W
Aradu.X6G84
Araip.17KGH
Max
Min
Araip.C36LC
Aradu.5N08M
Aradu.WZF00
(B)
Aradu.IX9G9
Araip.0B5Q5
Araip.4U4XR
FPKM
Aradu.6L1EK
Aradu.CA8XJ
Araip.MHR6K
Araip.K2CBN
Aradu.Z6I4Q
Araip.97C7E
Araip.V1VB7
Aradu.XEX7M
Aradu.LW8Y2
Aradu.5Q1VY
Araip.28KZQ
Aradu.K00WP
Araip.03YVC
Aradu.S0KU9
FPKM
Araip.PVV4Q
Araip.4C1AU
Aradu.K82B1
Aradu.L9W8G
Araip.UAK9J
Araip.438GZ
Araip.V8XWP
Aradu.C74HT
Aradu.WS4W4
(C)
Araip.WC91U
Araip.ZW9IZ
FPKM
Aradu.8QL4Z
Aradu.I01VW
Aradu.MHD5A
Aradu.8LY9S
Araip.088VH
Araip.RR7BI
Aradu.H2HAG
Araip.QRJ4E
Aradu.00WD1
Aradu.BY7GE
Araip.645H3
Aradu.JBM4A
FPKM
Aradu.GVR46
Araip.VB4DZ
Araip.YS8I7
Aradu.ZU7F1
Aradu.FG89G
Araip.R7CDW
Araip.AJ7AK
Araip.EJ673
Fig. S8. Differentially expressed MYB, bHLH, and WD40 factors between wsc and WT.
