# Supplementary figures and images for: Transcriptome and metabolome reveal redirection of flavonoids in a white testa peanut mutant
Source: BMC Plant Biol. 2020 Apr 15;20:161. doi: 10.1186/s12870-020-02383-7 (PMC7161308; doi:10.1186/s12870-020-02383-7)

## Slide 1
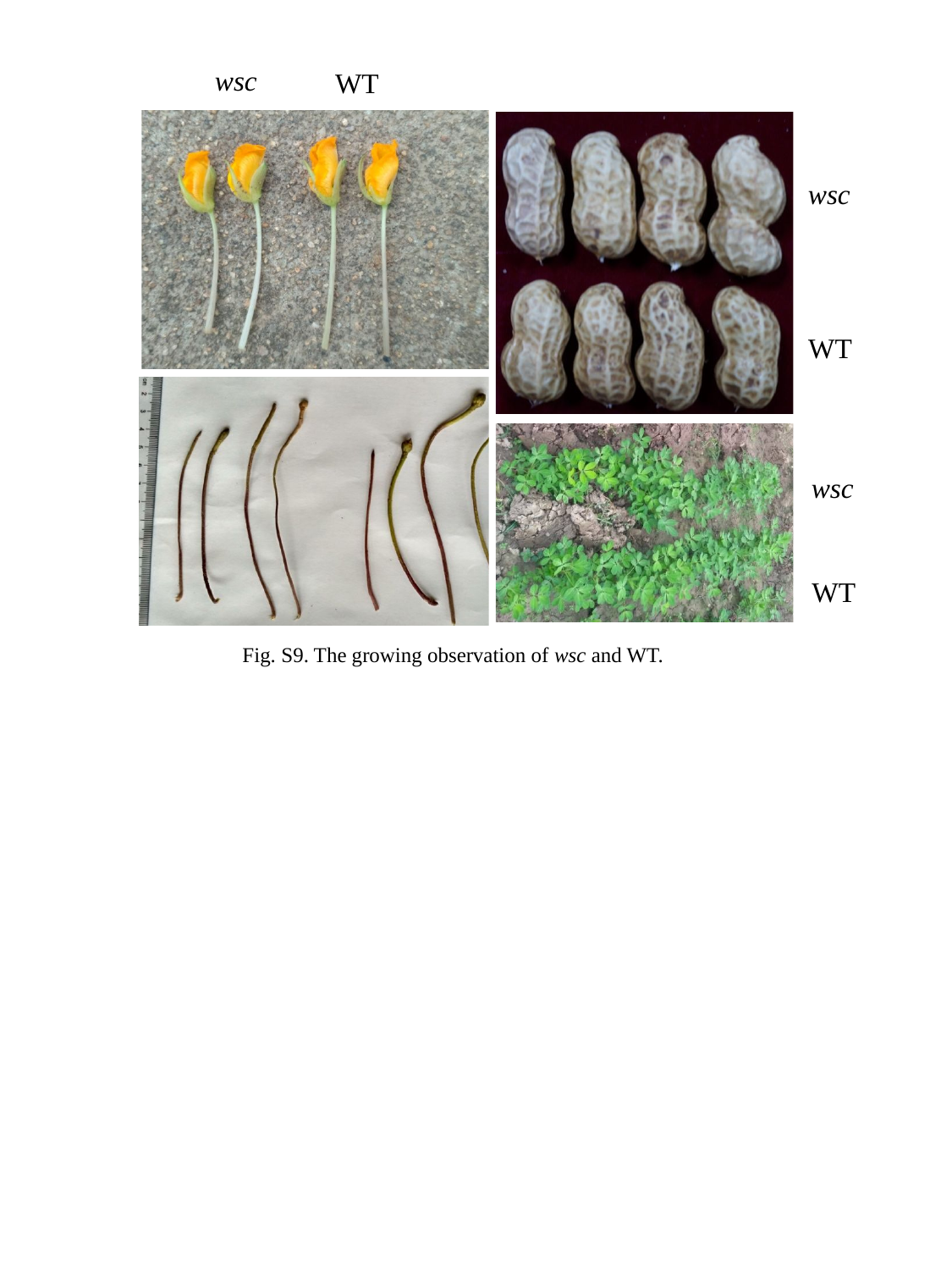

wsc
WT
wsc
WT
wsc
WT
Fig. S9. The growing observation of wsc and WT.

Supplement: Supplementary file 17 — Additional file 17. The growing observation of wsc and WT. [file 12870_2020_2383_MOESM17_ESM.ppt]

## Slide 1
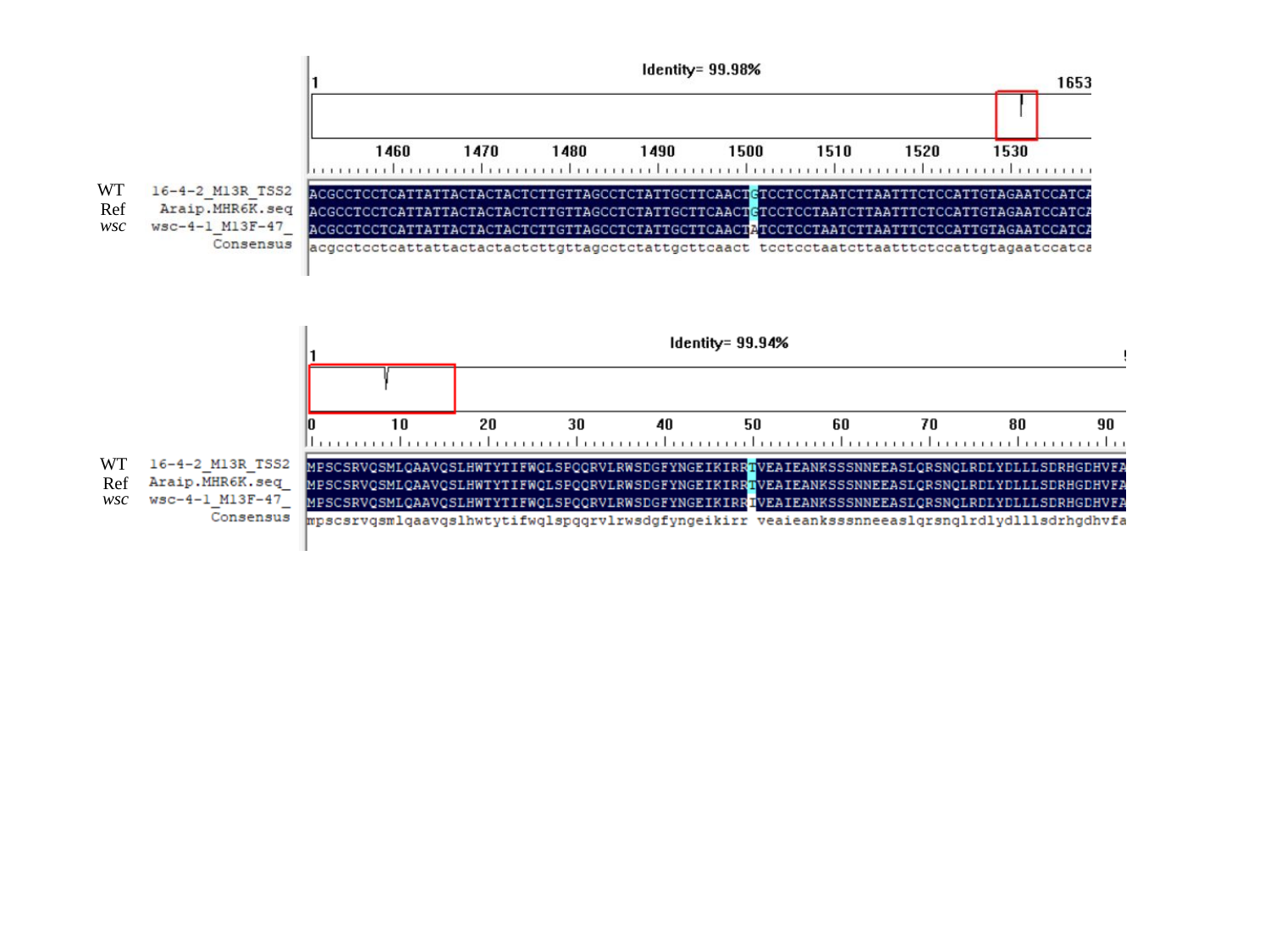

WT
Ref
wsc
WT
Ref
wsc

Supplement: Supplementary file 19 — Additional file 19. Sequence alignment of Araip.MHR6K between wsc and WT. [file 12870_2020_2383_MOESM19_ESM.ppt]
