# Supplementary material for: Transcriptome and metabolome reveal redirection of flavonoids in a white testa peanut mutant
Source: BMC Plant Biol. 2020 Apr 15;20:161. doi: 10.1186/s12870-020-02383-7 (PMC7161308; doi:10.1186/s12870-020-02383-7)
Supplement: Supplementary file 20 — Additional file 20. WSC losses affect multiple primary and secondly metabolism pathways. Mutant of WSC leads to a coordinated increase in transcript and metabolic levels of carbon metabolism pathways and hormone synthesis and signaling pathways while the flavonoids metabolism pathway was redirected. Up- and down-regulated genes and metabolites are shown in red and green, respectively. [file 12870_2020_2383_MOESM20_ESM.ppt]

## Slide 1
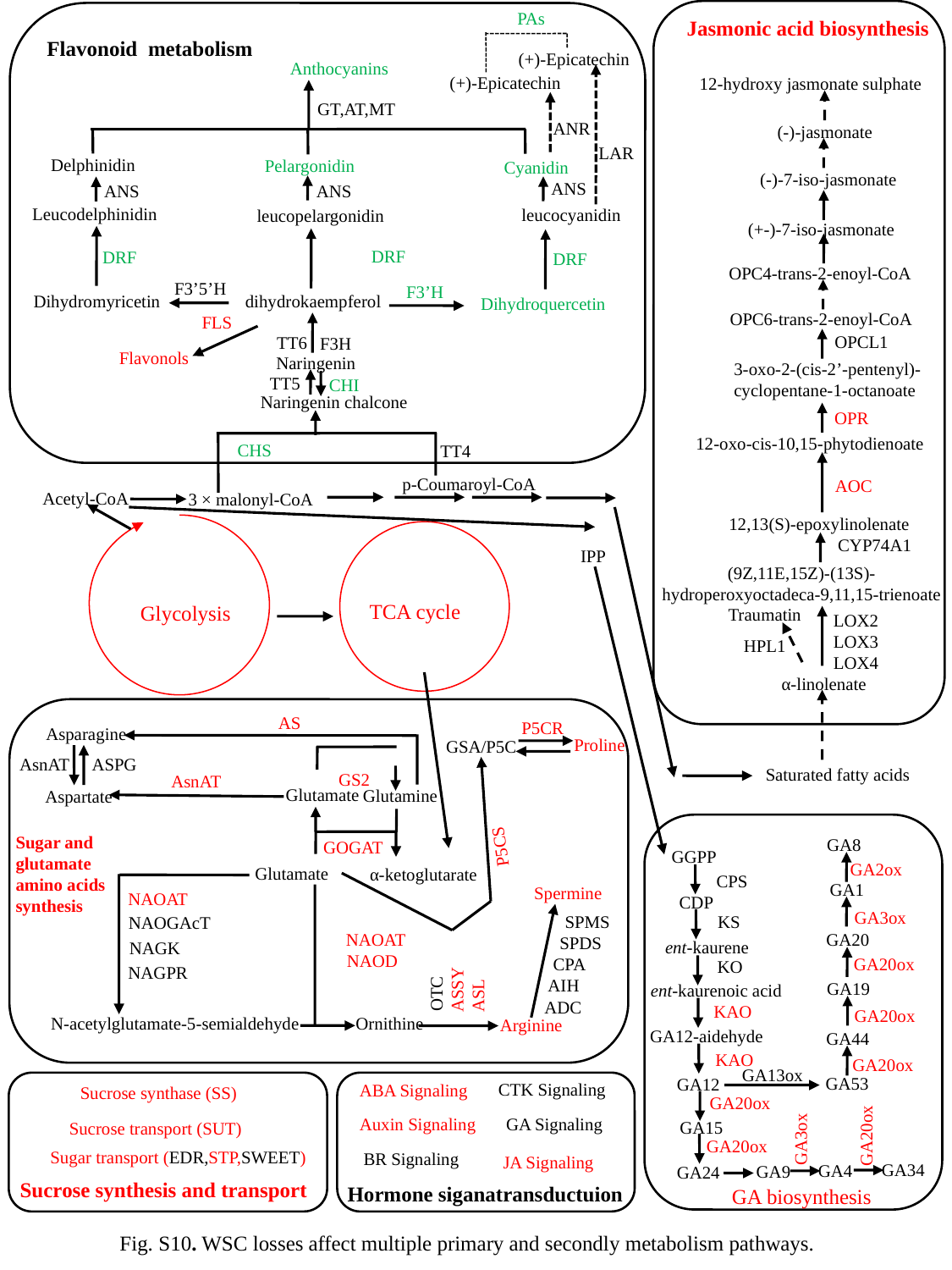

PAs
Flavonoid metabolism
(+)-Epicatechin
Anthocyanins
(+)-Epicatechin
GT,AT,MT
ANR
Delphinidin
Pelargonidin
Cyanidin
ANS
ANS
ANS
Leucodelphinidin
leucocyanidin
leucopelargonidin
DRF
DRF
DRF
F3’5’H
F3’H
Dihydromyricetin
dihydrokaempferol
Dihydroquercetin
FLS
TT6
F3H
Flavonols
Naringenin
TT5
CHI
Naringenin chalcone
CHS
TT4
Acetyl-CoA
3 × malonyl-CoA
Jasmonic acid biosynthesis
12-hydroxy jasmonate sulphate
(-)-jasmonate
(-)-7-iso-jasmonate
(+-)-7-iso-jasmonate
OPC4-trans-2-enoyl-CoA
OPC6-trans-2-enoyl-CoA
OPCL1
3-oxo-2-(cis-2’-pentenyl)-
cyclopentane-1-octanoate
OPR
12-oxo-cis-10,15-phytodienoate
AOC
12,13(S)-epoxylinolenate
CYP74A1
Traumatin
LOX2
LOX3
LOX4
HPL1
α-linolenate
Saturated fatty acids
LAR
p-Coumaroyl-CoA
IPP
(9Z,11E,15Z)-(13S)-hydroperoxyoctadeca-9,11,15-trienoate
TCA cycle
Glycolysis
P5CR
Proline
GSA/P5C
GS2
Glutamate
 Glutamine
P5CS
Sugar and glutamate amino acids synthesis
GOGAT
Glutamate
α-ketoglutarate
NAOAT
NAOGAcT
NAOAT
NAGK
NAOD
NAGPR
OTC
ASSY
ASL
N-acetylglutamate-5-semialdehyde
Ornithine
Arginine
AS
Asparagine
AsnAT
ASPG
AsnAT
Aspartate
GA8
GGPP
GA2ox
CPS
GA1
Spermine
CDP
GA3ox
SPMS
KS
GA20
SPDS
ent-kaurene
CPA
GA20ox
KO
AIH
GA19
ent-kaurenoic acid
ADC
KAO
GA20ox
GA12-aidehyde
GA44
KAO
GA20ox
GA13ox
GA53
GA12
CTK Signaling
ABA Signaling
Sucrose synthase (SS)
GA20ox
Auxin Signaling
GA3ox
GA Signaling
GA20ox
GA15
Sucrose transport (SUT)
GA20ox
Sugar transport (EDR,STP,SWEET)
BR Signaling
JA Signaling
GA34
GA4
GA9
GA24
Sucrose synthesis and transport
Hormone siganatransductuion
GA biosynthesis
Fig. S10. WSC losses affect multiple primary and secondly metabolism pathways.
